# FOXA1 regulates androgen receptor variant activity in models of castrate-resistant prostate cancer

**DOI:** 10.18632/oncotarget.4927

**Published:** 2015-08-13

**Authors:** Dominic Jones, Mark Wade, Sirintra Nakjang, Lewis Chaytor, James Grey, Craig N. Robson, Luke Gaughan

**Affiliations:** ^1^ Northern Institute for Cancer Research, Newcastle University, Newcastle Upon Tyne, NE2 4HH, UK

**Keywords:** prostate cancer, androgen receptor variants, FOXA1, transcriptional regulation

## Abstract

Retention of androgen receptor (AR) signalling in castrate-resistant prostate cancer (CRPC) highlights the requirement for the development of more effective AR targeting therapies. A key mechanism of resistance to anti-androgens is through expression of constitutively active AR variants (AR-Vs) that are refractory to next-generation therapies, including Enzalutamide and Abiraterone. By maintaining an androgenic gene signature, AR-Vs drive tumour survival and progression in castrate conditions. Critically, however, our understanding of the mechanics of AR-V-driven transcription is limited, particularly with respect to dependency on pioneer factor function. Here we show that depletion of FOXA1 in the CWR22Rv1 CRPC cell line abrogates the oncogenic potential of AR-Vs. Gene expression profiling reveals that approximately 41% of the AR-V transcriptome requires FOXA1 and that depletion of FOXA1 attenuates AR-V binding at a sub-set of analysed co-regulated genes. Interestingly, AR-V levels are elevated in cells depleted of FOXA1 as a consequence of attenuated negative feedback on the *AR* gene, but is insufficient to maintain cell growth as evidenced by marked anti-proliferative effects in FOXA1 knockdown cells. In all, our data suggests that AR-Vs are dependent on FOXA1 for sustaining a pro-proliferative gene signature and agents targeting FOXA1 may represent novel therapeutic options for CRPC patients.

## INTRODUCTION

Prostate cancer (PC) is the most prevalent male cancer in the Western world and represents the fourth most common cancer worldwide. PC growth is initially androgen-dependent hence the mainstay for treatment is hormone-ablation therapy using anti-androgens and/or androgen-deprivation therapies (ADT) [1, 2]. Unfortunately, in most patients, efficacy of these treatments is short-lived and the cancer typically recurs in a more aggressive form, termed castrate-resistant prostate cancer (CRPC), that remains challenging to treat and is largely fatal [3]. The development of second generation anti-androgen therapies, such as Enzalutamide [4, 5] and abiraterone [6, 7], have shown promise in the treatment of CRPC, but are not effective in all patients and the development of resistance has limited their success in the clinic [8, 9].

The androgen receptor (AR) is a member of the nuclear hormone receptor family of transcription factors that transmits androgenic signals to drive prostate growth and cellular transformation. Like other nuclear receptors, the process of AR-mediated transcription is complex and is tightly orchestrated within the nucleus by a plethora of co-regulatory proteins, a number of which directly acetylate and methylate the receptor to control the strength and duration of androgenic signalling [10 13]. Our understanding of the global functionality of the AR as a DNA-binding transcription regulator has been re-defined over the past number of years with the demonstration that additional transcription factors, such as FOXA1, control AR-chromatin deposition and are instrumental in controlling receptor-driven tissue-specific transcriptional programmes [14, 15]. Several independent studies have shown considerable overlap between ligand-inducible AR and FOXA1 DNA-binding sites within both proximal promoter and distal enhancer elements of target genes suggesting a pioneering role for FOXA1 in facilitating receptor-mediated gene expression [16–18]. Intriguingly, however, depletion of FOXA1 in several PC cell lines is not deleterious to all AR binding events at shared androgen response element (ARE) and FOXA1-binding sites, and a concurrent stimulation of receptor binding to new AREs within the genome suggest that FOXA1 may function to both facilitate and repress AR signalling at discriminate genomic loci [16, 19].

Several aberrations in the AR signalling cascade have been identified in CRPC, including AR mutation and non-androgenic activation of the receptor, that facilitate AR activity in castrate conditions and contribute to conventional and next-generation anti-androgen treatment failure [20, 21]. More recently, the identification of novel AR variants in CRPC that are refractory to hormonal therapies has provided an additional avenue for treatment evasion and progression to advanced disease [22, 23]. AR variants (termed AR-Vs), such as the clinically-relevant AR-V7 protein, lack the conventional C-terminal ligand-binding domain (LBD), but retain the potent N-terminal transactivation domain and DNA-binding domain, and are thus capable of driving the androgenic signalling programme in castrate conditions and remain unchallenged by the current repertoire of receptor-targeting agents [24, 25]. Moreover, although splicing aberrations have been shown to be responsible for the generation of several AR-Vs in PC cell-line models, such as CWR22Rv1 and VCaP, and patient samples of CRPC [26, 27], there is clear evidence that genomic deletion of exons coding the LBD is an additional mechanism of AR-V production in advanced disease [28]. Importantly, both experimental and translational analyses indicates overexpression of AR-Vs in approximately 50%–60% of CRPC patients with the figure rising further in metastatic disease [29].

Our understanding of how AR-Vs are regulated remains limited, particularly with respect to their dependency on pioneer factors for transcriptional activity. To this end, we assessed the role of FOXA1 in regulation of AR-Vs in CWR22Rv1 cells and show that 41% of the AR-V transcriptome overlaps with a FOXA1-dependent gene signature. Consistent with previous findings in LNCaP cells [19], depletion of FOXA1 in CWR22Rv1 cells up-regulates several genes, including *PSA* and *KLK2*, that is a direct consequence of elevated AR-V levels. Irrespective of this, however, FOXA1 knockdown markedly reduces cell growth and is shown to be an important regulator of the pro-proliferative function of AR-Vs in CRPC. In all, our data supports the notion that AR-V activity is regulated by pioneer factors akin to the full-length AR (FL-AR), and FOXA1 may represent a suitable target for therapy in advanced disease.

## RESULTS

### AR-Vs are constitutively chromatin bound and refractory to enzalutamide

**Figure 1:**
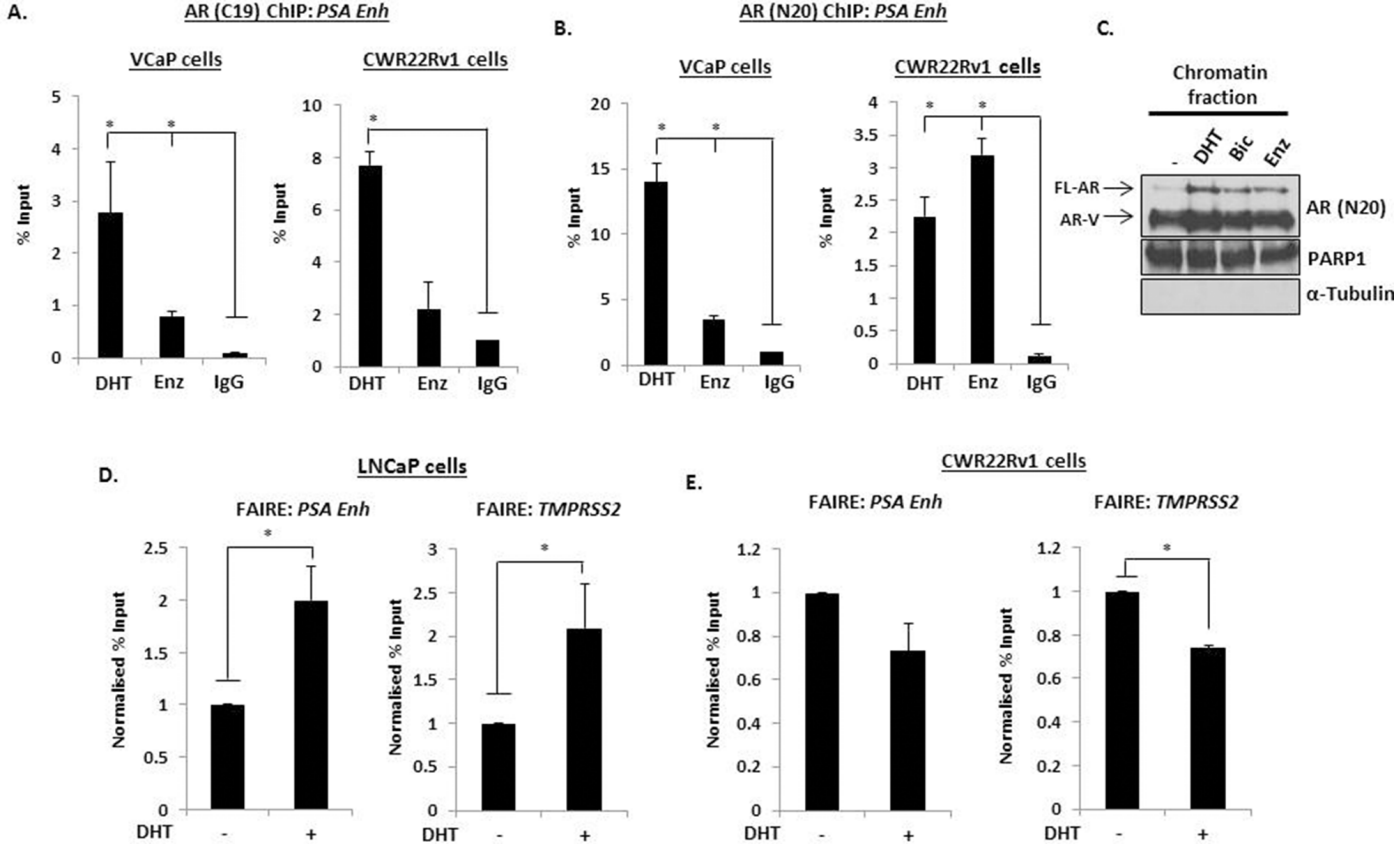
AR-Vs are constitutively chromatin bound Chromatin immunoprecipitation (ChIP) was performed using AR (C-19) **A.** or AR (N-20) **B.** antibodies in VCaP and CWR22Rv1 cells treated with 10 nM DHT or 1 μM Enzalutamide for 4 hours and AR recruitment to the *PSA* enhancer element was analysed by quantitative PCR. **C.** Chromatin fractionation was performed in CWR22Rv1 cells treated as above and resultant samples were subject to anti-AR, PARP1 (chromatin) and α-tubulin (cytoplasmic) antibodies. Formaldehyde-assisted isolation of response elements (FAIRE) was performed in LNCaP **D.** and CWR22Rv1 **E.** cells after 4 hours 10 nM DHT treatment and resultant DNA was analysed by quantitative PCR using primers specific to the indicated genes. All data represents the mean of at least three independent experiments +/− SE (*denotes *p*-value < 0.05)

The ability for AR-Vs to confer resistance to current and next-generation ADT and anti-androgens is well established and is likely a result of constitutive activation of an androgenic signalling programme refractory to AR-targeted agents [27]. However, our understanding of the chromatin-binding kinetics and transcriptional requirements of AR-Vs in CRPC is very limited. To this end, we firstly assessed the recruitment of AR-Vs to several known AR target genes in CWR22Rv1 cells, that express very high levels of receptor variants, and compared binding profiles to that of the FL-AR in both CWR22Rv1 and VCaP cells. Consistent with the effect of Enzalutamide as a LBD-targeting agent [30], chromatin immunoprecipitation (ChIP) experiments, using a C-terminal-targeting AR antibody (C-19), demonstrate that FL-AR is depleted from the *PSA* enhancer in both VCaP and CWR22Rv1 cells (Figure 1A). Importantly, however, an antibody targeting an N-terminal receptor epitope (AR N-20) shows that chromatin binding of the AR is retained upon both Enzalutamide treatment and specific depletion of FL-AR in CWR22Rv1 cells suggesting AR-variants are constitutively bound to chromatin (Figure 1B and Supplementary Figure S1). In contrast, although VCaP cells express a population of AR-Vs, the levels are considerably lower than CWR22Rv1 cells and this is reflected in the marked reduction in AR binding at the PSA enhancer in response to anti-androgen treatment.

To confirm constitutive AR-V-DNA binding interaction, chromatin extractions were performed in CWR22Rv1 cells grown in the presence and absence of 10 nM DHT with and without 1 μM Bicalutamide or Enzalutamide. As shown in Figure 1C, levels of chromatin-bound FL-AR are elevated upon DHT stimulation and reduced upon treatment with anti-androgens. In keeping with the ChIP data, however, AR-Vs were found to be constitutively chromatin bound irrespective of hormonal or anti-androgen status suggesting that AR-Vs have the potential to retain androgenic signalling by mediating robust DNA-binding activity in cells. This finding was supported further using formaldehyde-assisted isolation of response elements (FAIRE) in both FL-AR-expressing LNCaP cells and CWR22Rv1 cells. As shown in Figure 1D, unlike LNCaP cells which displayed increased chromatin relaxation at *cis*-regulatory elements within the *PSA* enhancer and *TMPRSS2* promoter in response to DHT treatment that reflects recruitment of FL-AR to these loci, AR-V expressing CWR22Rv1 displayed a consistently open chromatin conformation in the absence of androgen indicating the presence of a constitutively-bound AR-V population (Figure 1E). Intriguingly, in response to DHT treatment, *cis*-regulatory elements of *PSA* and *TMPRSS2* genes was compacted by hormone treatment suggesting that activation of FL-AR may counteract chromatin opening by AR-Vs although this needs to be fully evaluated.

### FOXA1 depletion elevates PSA, KLK2 and AR-V mRNA in CWR22Rv1 cells

**Figure 2:**
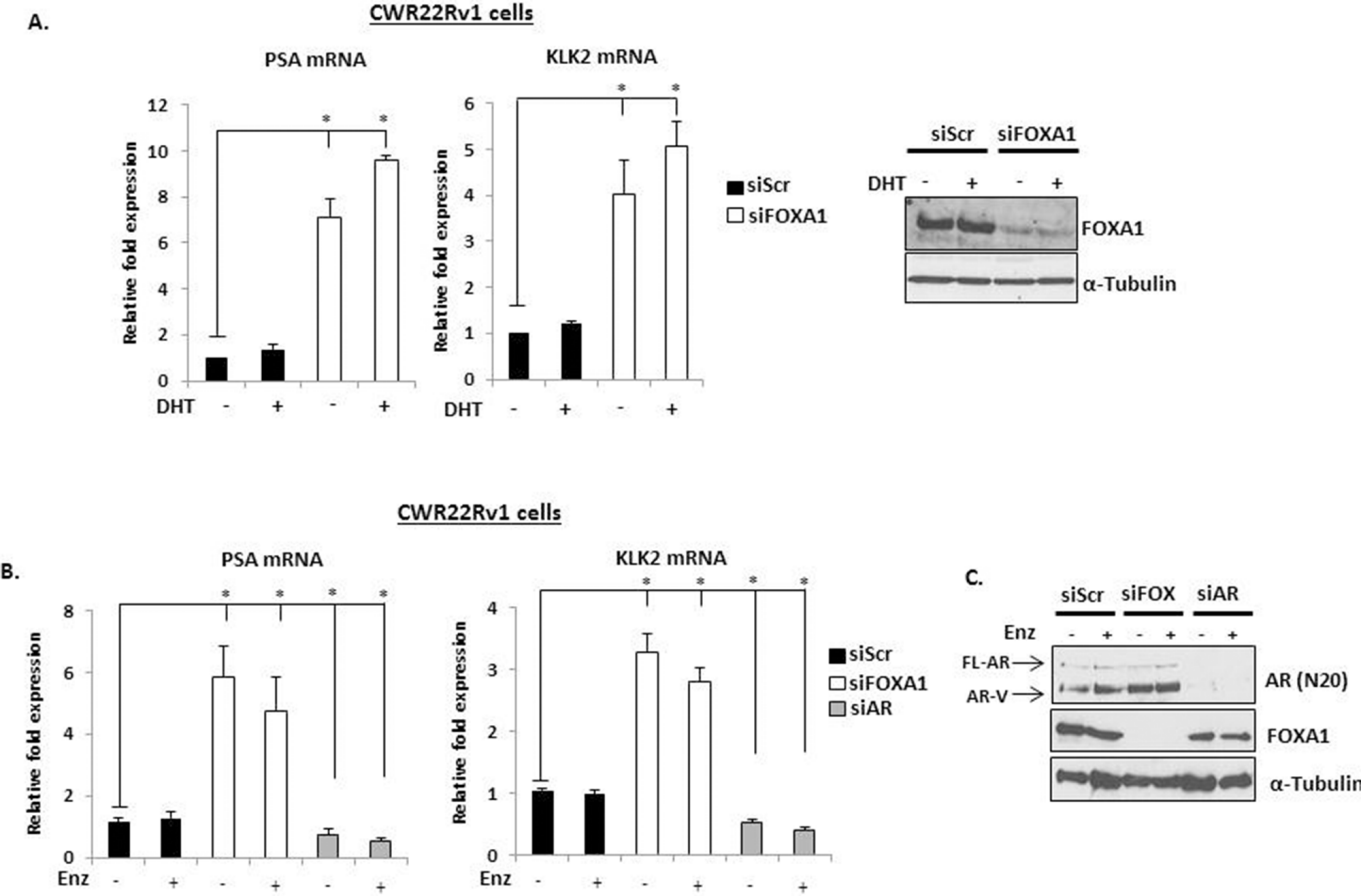
FOXA1 depletion up-regulates *PSA* and *KLK2* expression in CWR22Rv1 cells **A.** FOXA1 levels were reduced by siRNA knockdown for 48 hours in CWR22Rv1 cells and expression of *PSA* and *KLK2* was measured after 24 hours 10 nM DHT stimulation by quantitative PCR. Representative FOXA1 protein levels are shown on the right. **B.** As in **(A)**, but with the inclusion of a 1 μM Enzalutamide treatment arm for 24 hours prior to quantitative analysis. **C.** AR and FOXA1 knockdown is confirmed by immunoblotting using anti-AR and –FOXA1 antibodies. All data represents the mean of at least three independent experiments +/− SE (*denotes *p*-value < 0.05)

Given that FOXA1 is instrumental in regulating FL-AR chromatin deposition at discriminate androgenic cistromes [16, 19, 31], it is conceivable that FOXA1 may co-operate with AR-Vs to facilitate their role as oncogenic drivers in advanced PC. Our demonstration that AR-Vs are constitutively chromatin bound in CWR22Rv1 cells provided an indication that interplay between FOXA1 and receptor variants may exist. We therefore firstly assessed the impact of FOXA1 depletion on the AR-target genes *PSA* and *KLK2* in CWR22Rv1 cells as both have been shown to be regulated by the pioneer factor in PC cells [19] As expected, *PSA* and *KLK2* expression was refractory to 10 nM DHT stimulation due to their transcription being largely driven by AR-Vs (Figure 2A and Supplementary Figure S2). Importantly, FOXA1 knockdown markedly elevated expression of both *PSA* and *KLK2* in the presence and absence of 1 nM and 10 nM DHT, a finding that is consistent with reports describing FOXA1 as a regulator of FL-AR activity [19]. Furthermore, Enzalutamide treatment failed to reduce *PSA* and *KLK2* expression suggesting that this phenomenon was driven by AR-Vs; a finding supported by receptor knockdown (Figure 2B).

**Figure 3:**
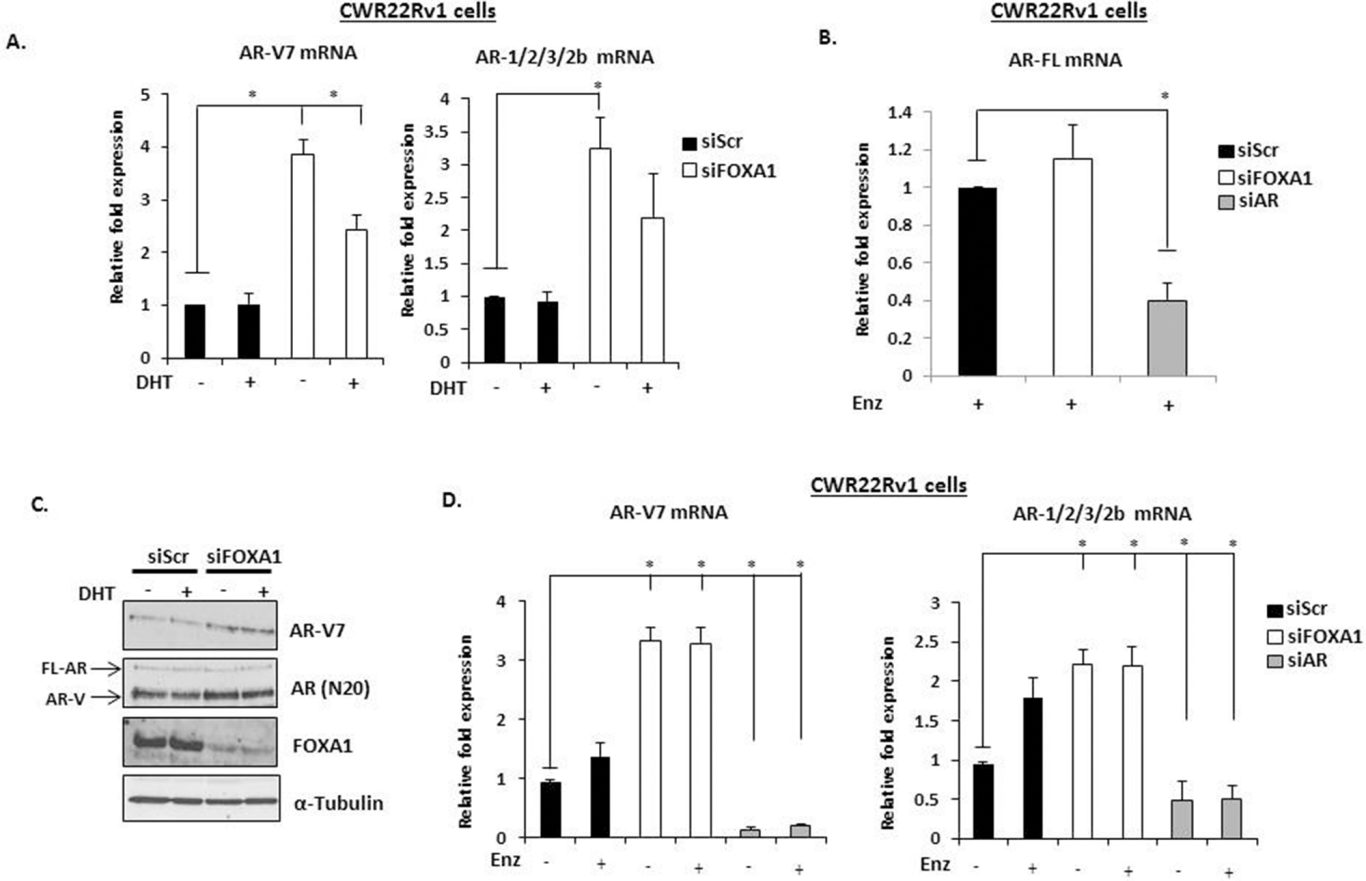
FOXA1 knockdown elevates AR-V expression in CWR22Rv1 cells **A.** FOXA1 was depleted by siRNA for 48 hours in CWR22Rv1 cells and levels of AR-V7, AR-1/2/3/2b were measured after 24 hours 10 nM DHT stimulation by quantitative PCR. **B.** As in (A), but cells were additionally depleted of AR by siRNA and treated for 24 hours with Enzalutamide prior to analysis of FL-AR mRNA. **C.** Western analysis of FOXA1 knockdown samples using AR (N-20), AR-V7, FOXA1 and α-Tubulin antibodies. **D.** As in (A) but with the inclusion of a 1 μM Enzalutamide treatment arm. All data represents the mean of at least three independent experiments +/− SE (*denotes *p*-value < 0.05)

Given that both AR target genes were overexpressed in conditions where only AR variants are likely to be functional, we hypothesised that FOXA1 regulated expression of these receptor isoforms. As shown in Figure 3, FOXA1 depletion increased mRNA expression of both AR-V7 and AR-1/2/3/2b in CWR22Rv1 cells (Figure 3A), but not the FL-AR (Figure 3B) and this was confirmed at the protein level using anti-AR-V7 and AR (N-20) antibodies (Figure 3C). Importantly, increased AR variant levels persisted in the presence of Enzalutamide (Figure 2C and Figure 3D) and is consistent with a role for the AR-Vs in driving up-regulation of *PSA* and *KLK2* in FOXA1 knockdown cells. This data was confirmed using additional FOXA1-targeting siRNAs (Supplementary Figures S3 and S4).

### FOXA1 regulates AR-V expression through an *AR* gene repressor element

**Figure 4:**
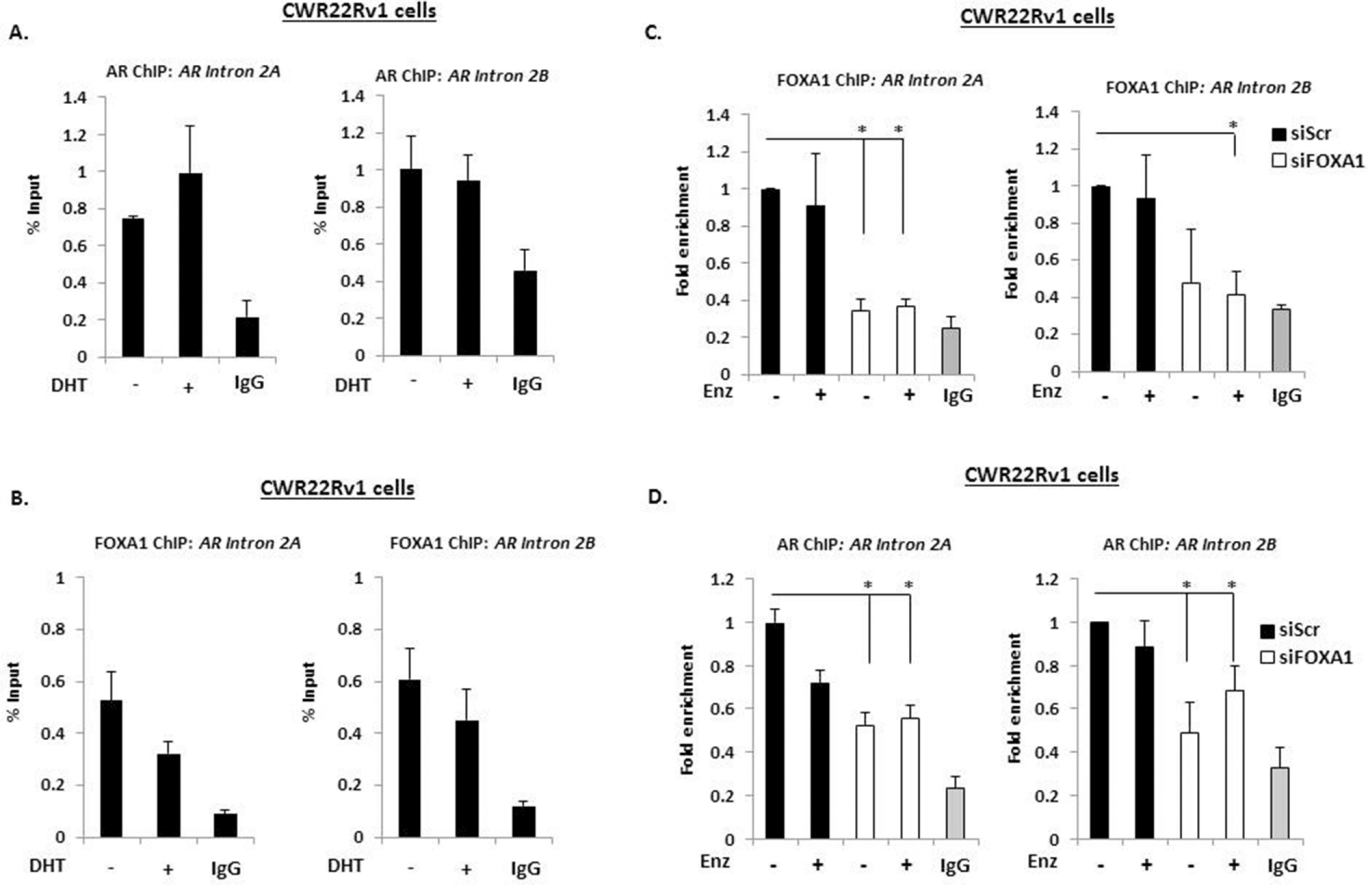
A downstream repressive element in the AR gene controls AR-V expression Chromatin immunoprecipitation (ChIP) experiments in CWR22Rv1 cells using AR (N-20) **A.** and FOXA1 **B.** antibodies to assess recruitment at intron 2 of the *AR* gene in response to 4 hour 10 nM DHT treatment. CWR22Rv1 cells depleted of FOXA1 were treated with Enzalutamide for 4 hours and subject to ChIP using FOXA1 **C.** and AR **D.** antibodies to assess recruitment to intron 2 of the *AR* gene. Fold enrichment is calculated in **(D)** to demonstrate loss of AR recruitment upon FOXA1 depletion (*denotes *p* = < 0.05 significance between siScr control and FOXA1 depletion). All data represents the mean of at least three independent experiments +/− SE (*denotes *p*-value < 0.05)

The existence of an androgenic downstream repressor element (DRE) in intron 2 of the *AR* gene has been shown to control receptor expression in VCaP cells [32]. By recruiting the histone methyltransferase LSD1 to this loci in response to DHT, the receptor down-regulates *AR* gene transcription through demethylating histone H3 lysine 4 at upstream *cis*-regulatory elements. As shown in Supplementary Figure S5A, we have confirmed that active FL-AR is recruited to two regions of the DRE (Intron 2A and 2B) in VCaP cells that is attenuated in the presence of Enzalutamide. Moreover, exposure of VCaP cells to 10 nM DHT markedly reduced AR-V7 levels (Supplementary Figure S5B), indicating that AR variant expression is also under the control of this phenomenon and is consistent with recently published data [33]. Although not as robust as VCaP cells, reduced AR-V7 and AR-1/2/3/2b expression in response to DHT was also demonstrated in CWR22Rv1 cells depleted of FOXA1 (Figure 3A), suggesting the same auto-regulatory loop exists for AR-V regulation in an additional model of advanced CRPC. From these indications, we reasoned that AR-V expression in CWR22Rv1 cells is self-limiting and FOXA1 may function to facilitate AR deposition at this DRE and down-regulate total AR mRNA levels. Therefore, we first sought to establish if the DRE bound both AR and FOXA1 in CWR22Rv1 cells. ChIP experiments using the AR (N-20) antibody demonstrated enrichment of receptor species above IgG control even in the absence of DHT (Figure 4A) suggesting that AR-Vs are constitutively bound to this site, but not to a control intron 2 region (Supplementary Figure S6A). Consistent with AR binding, FOXA1 was enriched at the DRE (Figure 4B) albeit to lower levels than the *PSA* promoter (Supplementary Figure S6B) and was not affected by Enzalutamide (Figure 4C). To assess the role of FOXA1 in regulating AR-V recruitment to the *AR* gene DRE, we depleted FOXA1 in CWR22Rv1 cells and demonstrated that this markedly reduced both FOXA1 and AR-V enrichment at the repressive *cis*-regulatory element (Figures 4C and 4D), and the *PSA* promoter (Supplementary Figure S7A), but not at a control DRE element (Supplementary Figure S7B). Our findings demonstrate pertinent interplay between FOXA1 and AR-Vs for controlling receptor variant expression and provide a mechanistic insight into the observed elevation of AR-Vs in response to pioneer factor knockdown in CRPC.

### FOXA1 regulates AR-V activity

In order to establish a role for FOXA1 in regulating global AR-V transcriptional activity, we individually depleted AR and FOXA1 in CWR22Rv1 cells grown in the presence of Enzalutamide and assessed differential gene expression signatures in both experimental arms. To confirm that the effect of AR knockdown was a specific read-out for AR-V function, we firstly conducted analysis on CWR22Rv1 cells grown in steroid-depleted conditions with and without Enzalutamide to assess the activation status of FL-AR in these conditions. Importantly, we found that maintaining cells in the absence of DHT for 72 hours was sufficient to completely abrogate FL-AR activity as treatment with Enzalutamide failed to impact on global gene expression (data not shown). We therefore reasoned that all genes found to be de-regulated upon AR knockdown can be attributed to AR-V activity. In support of this, Figure 1C demonstrates low levels of FL-AR bound to chromatin in androgen-depleted conditions suggesting compromised FL-AR signalling.

**Figure 5:**
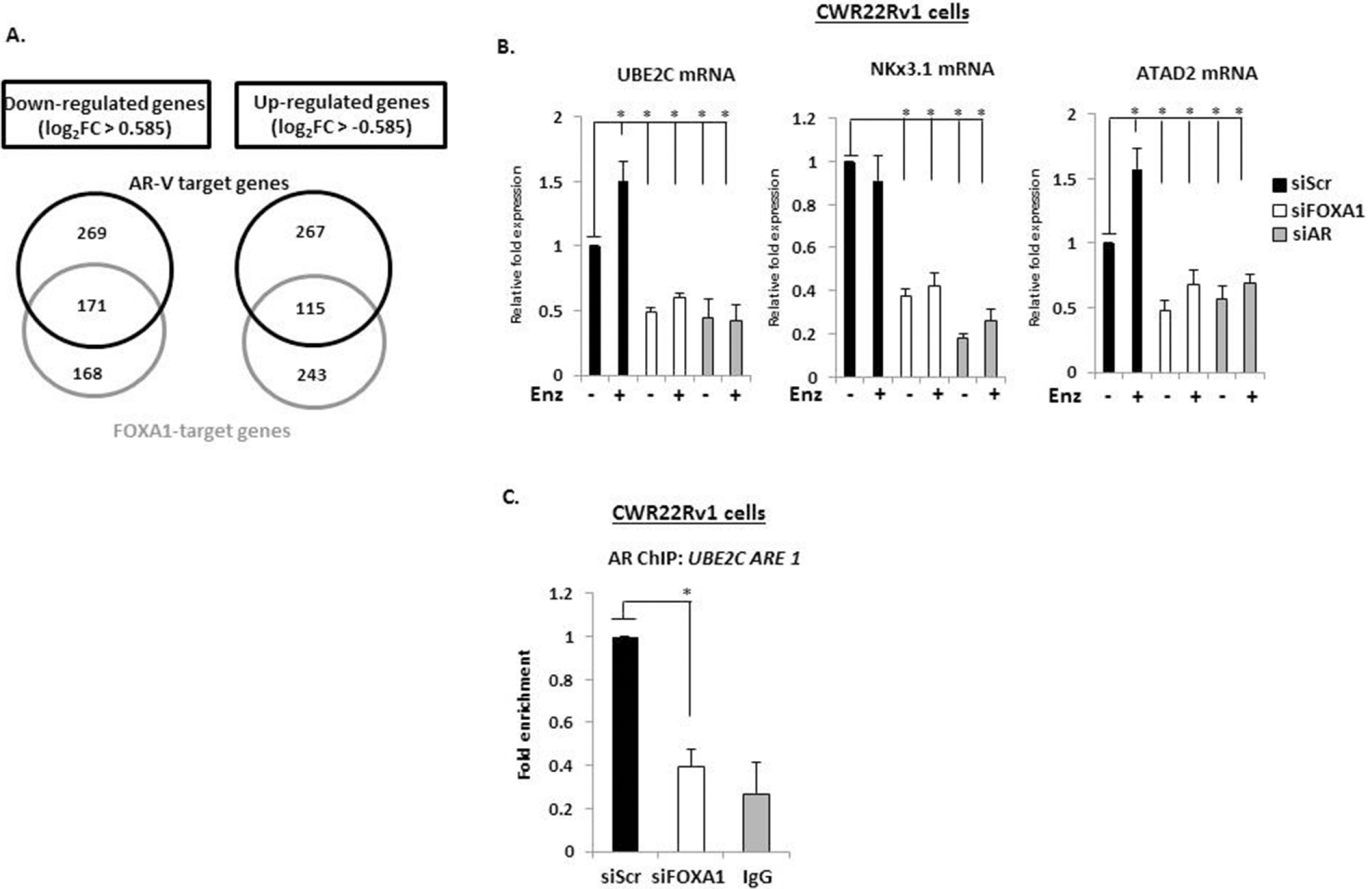
AR-V- and FOXA1-target genes show considerable overlap **A.** Venn diagrams of genes demonstrating 1.5-fold up- and down-regulation in response to AR and FOXA1 depletion in CWR22Rv1 cells grown in steroid-depleted conditions + 1 μM Enzalutamide. **B.** Validation of *UBE2C*, *NKx3.1* and *ATAD2* as AR-V- and FOXA1-co-regulated genes using quantitative PCR analysis of CWR22Rv1 cells depleted of AR or FOXA1 treated with and without Enzalutamide for 24 hours. **C.** Chromatin immunoprecipitation (ChIP) in CWR22Rv1 cells depleted of FOXA1 using an AR (N-20) antibody to assess AR-V binding to the *UBE2C* promoter (*denotes *p* = < 0.05 significance between siScr control and FOXA1 depletion). All data represents the mean of at least three independent experiments +/− SE (*denotes *p*-value < 0.05)

Depletion of AR and FOXA1 resulted in differential expression of respective 3085 and 2224 probes (Supplementary Tables S1 and S2), equating to 2366 and 1722 annotated genes (Supplementary Figure S8) with 41% of AR-V target genes demonstrating overlap with FOXA1; and 56% of the FOXA1 transcriptome being dually controlled by AR-Vs. Of those genes demonstrating a significant 1.5-fold up- (382 genes) and down-regulation (440 genes) in response to AR-V depletion, 30% and 39% respectively, were also found to be similarly up- and down-regulated by FOXA1 (Figures 5A), suggesting a considerable overlap in functionality of the two proteins in AR-V-mediated signalling.

Several studies have suggested AR-Vs regulate a distinct gene-set to that of FL-AR; including a 25 gene signature, including *UBE2C*, that was defined as being specifically up-regulated by ectopically-expressed AR-V7 in LNCaP cells (termed AR-V7 UP, [34]). Integrating our AR-V-activated transcriptome data (siAR DOWN) with this LNCaP-derived AR-V7 UP signature identified an overlap of 20 out of 25 genes (80%) (Supplementary Figures S9A and S9C) suggesting concordant activity of variants in distinct cellular backgrounds. Additionally, we identified 13 out of 25 (52%) AR-V7 UP genes to be regulated by AR-V and FOXA1 in CWR22Rv1 cells (Supplementary Figures S9B and S9C) supporting the concept that pioneer activity is important for variant-mediated transcription.

A recent and more physiological study identified a total of 285 non-duplicated probes to be up-regulated by AR-Vs in CWR22Rv1 cells [35]. Comparison with our AR-V signature demonstrated an overlap of 20% (Supplementary Table S3), and importantly, 49% of these genes were co-regulated by FOXA1 indicating a robust overlap in functionality of the pioneer factor and receptor variants (Supplementary Table S4).

To further validate FOXA1 as a regulator of AR-V activity, we focussed on the genes *UBE2C*, *NKx3.1* and *ATAD2* that were identified from the micro-array as being down-regulated in response to FOXA1 and AR knockdown. As shown in Figure 5B and Supplementary Figure S10, each gene was robustly down-regulated in response to FOXA1, AR and AR-V depletion using specific siRNA oligonucleotides and this occurred irrespective of DHT dose (Supplementary Figure S11). Interestingly, *UBE2C* and *ATAD2* expression was modestly elevated in response to Enzalutamide, suggesting that FL-AR may act to repress the function of AR-Vs at these genes (Figure 5B). ChIP experiments in CWR22Rv1 cells depleted of FOXA1 showed robust reduction in AR-V recruitment to a *cis*-regulatory element of the *UBE2C* gene indicating dependency of FOXA1 for receptor variant target gene recruitment and transcriptional output (Figure 5C). To confirm that the predominant AR species at the these promoter elements are AR-Vs, we specifically depleted FL-AR using an exon 4-targeting siRNA and demonstrated no loss of receptor retention (Supplementary Figure S12). Intriguingly, the effect of attenuated AR-V promoter association is not recapitulated globally; chromatin extractions from CWR22Rv1 cells depleted of FOXA1 demonstrate increased chromatin association of AR-Vs, and specifically AR-V7, that is likely a consequence of elevated protein levels in FOXA1 knockdown cells (Supplementary Figure S13).

### FOXA1 regulates pro-proliferative activity of AR-Vs

**Figure 6:**
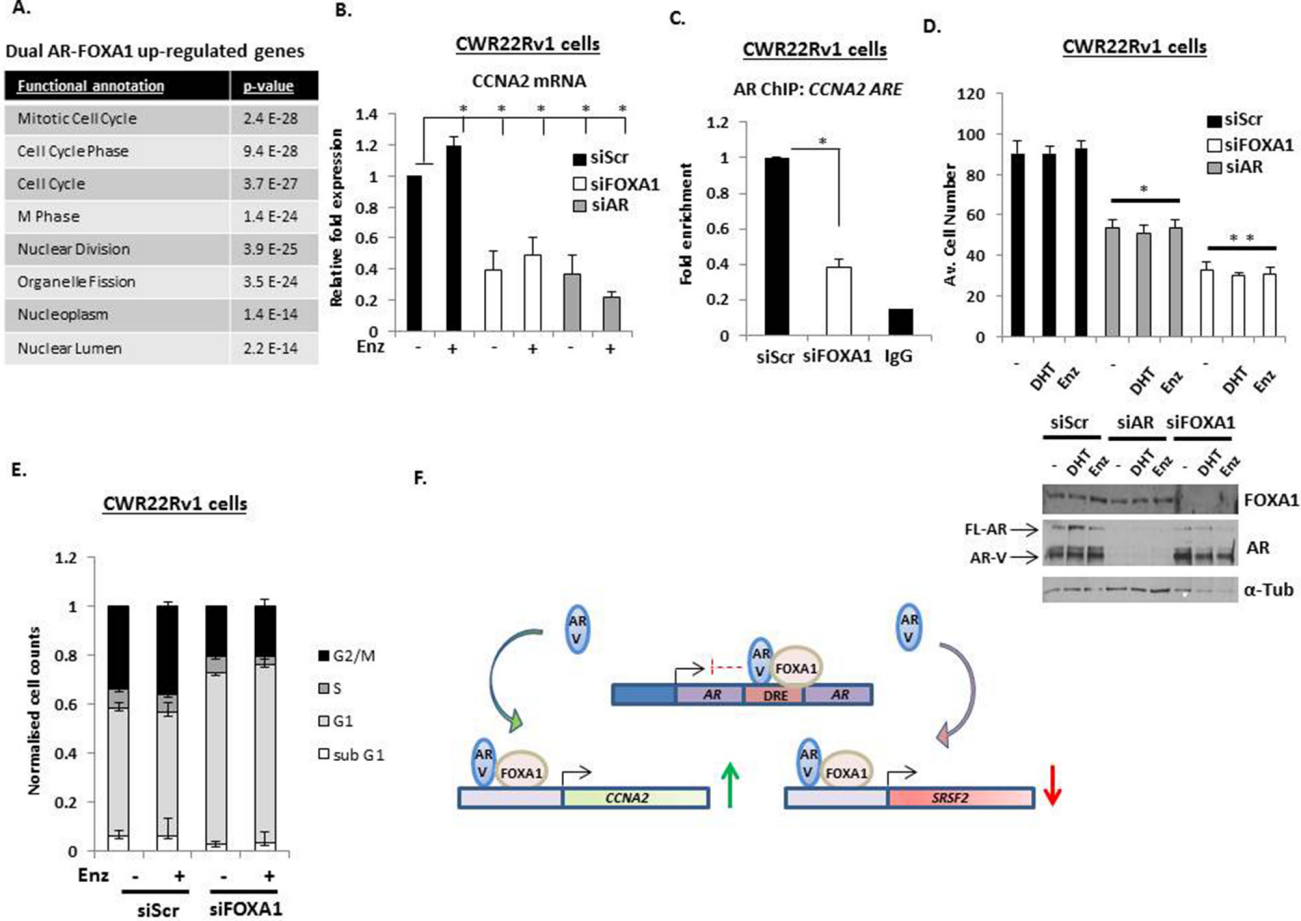
AR-Vs and FOXA1 co-regulate pro-proliferative genes **A.** Gene ontology analysis of co-regulated AR- and FOXA1-target genes shows significant enrichment of cell cycle and cell division associated genes. **B.** Impact of FOXA1 and AR knockdown on C*yclin A2* (*CCNA2*) expression in CWR22Rv1 cells grown in steroid-depleted media treated with 10 nM DHT or 1 μM Enzalutamide as measured by quantitative PCR. **C.** Chromatin immunoprecipitation (ChIP) was performed in CWR22Rv1 cells depleted of FOXA1 to assess AR-V recruitment to the *CCNA2* promoter (*denotes *p* = < 0.05 significance between siScr control and FOXA1 depletion). **D.** Proliferation assays to assess effect of AR or FOXA1 knockdown in CWR22Rv1 cells treated with 10 nM DHT or 1 μM Enzalutamide for 96 hours (* denotes *p* = < 0.05; ** denotes *p* = < 0.01 significance between siScr control and AR or FOXA1 knockdown, respectively). Representative western blots indicate AR and FOXA1 levels in response to protein knockdown. **E.** Cell cycle analysis using propidium iodide (PI) flow cytometry in CWR22Rv1 cells depleted of FOXA1 grown in the presence and absence of 1 μM Enzalutamide for 72 hours. All data represents the mean of at least three independent experiments +/− SE (*denotes *p*–value < 0.05). **F.** Diagrammatic representation of FOXA1-AR-V interplay in CRPC. Expression of AR-Vs are regulated by FOXA1 at the downstream repressive element (DRE) of the *AR* gene. FOXA1 also facilitates respective androgen-independent activation and repression of genes, such as *CCNA2* and *SRSF2* by AR-Vs.

Applying gene set enrichment (GSE) analysis (using Database for Annotation, Visualization and Integrated Discovery (DAVID)) to our AR-V and FOXA1-co-regulated genes identified cell cycle control as the highest-ranked pathways dually controlled by AR-V and FOXA1 (Figure 6A). *Cyclin A2* (*CCNA2*), *Cyclin B1* (*CCNB1*), *Cyclin E2* (*CCNE2*) and *Cyclin-dependent Kinase 1 (CDK1)* were all found to be significantly down-regulated in response to individual depletion of AR and FOXA1, indicating a role for FOXA1 in controlling the activity of AR-Vs at pro-proliferative genes in CRPC. To confirm this, *CCNA2* expression was assessed in CWR22Rv1 cells depleted of either FOXA1, AR or AR-Vs in the presence and absence of Enzalutamide. As shown in Figure 6B, *CCNA2* mRNA levels were unaffected by Enzalutamide treatment, indicating a dependency on AR-V-mediated transcription for *CCNA2* expression, that was confirmed by AR-V knockdown (Supplementary Figure S14). Importantly, FOXA1 depletion attenuated *CCNA2* expression to levels equivalent to AR knockdown confirming our micro-array data. Moreover, using ChIP, we found that FOXA1 depletion diminished AR-V association at the *CCNA2* promoter (Figure 6C) indicating the importance of FOXA1 as a pioneer factor for AR-V function at this loci.

Based on the results of our GSEA of shared AR-V/FOXA1 genes, we next assessed the effect of AR-V and FOXA1 depletion on proliferation of CWR22Rv1 cells grown in the presence and absence of DHT and Enzalutamide. Consistent with the role of AR-Vs in driving CWR22Rv1 cell growth [24], DHT and anti-androgen treatment failed to effect proliferation of control transfected cells (siScr), while AR knockdown resulted in approximately 50% reduction in cell proliferation (Figure 6D). In keeping with the role of FOXA1 as a pioneer factor for AR-Vs, depletion of the FOXA1 protein down-regulated cell proliferation by over 60% suggesting that in addition to regulating the pro-proliferative activity of AR-Vs, FOXA1 may also control genes outside of the AR signalling cascade that are drivers of cell turnover. Using cell-cycle analysis, we found that FOXA1 depletion caused a cytostatic effect in CWR22Rv1 cells in which G1 phase is markedly elevated at the expense of S-, G_2/_M- and sub-G1- phases of the cell cycle (Figure 6E). In all, our data demonstrates that FOXA1 is a key regulator of AR-Vs in CRPC and supports the concept of targeting pioneer factor function as a treatment for advanced PC (see Figure 6F for diagrammatic representation of FOXA1-mediated regulation of AR-Vs in CRPC).

## DISCUSSION

The development of new strategies to inactivate the AR signalling cascade in advanced CRPC is essential. The selection of cells displaying aberrant AR activity during first-line hormonal therapy that enable receptor function in castrate-like conditions is a hallmark of treatment failure and disease progression [1]. For example, mutation and non-androgenic stimulation of the AR enable persistence of receptor function in the presence of targeted therapy, such as the anti-androgen bicalutamide [21]. The development of the second generation anti-androgen Enzalutamide has had success in the clinic for treatment of CRPC, but efficacy is limited, in part, by the of appearance of an AR_F876L_ mutation in the LBD that converts Enzalutamide to an agonist [9, 36]. Importantly, the complexity of first- and second-generation therapy resistance has increased with the key finding that alternative, constitutively active forms of the AR that lack the LBD, termed AR-Vs, are over-expressed in CRPC and hence are refractory to current anti-androgens [8, 29]. Indeed, detection of AR-Vs in circulating CRPC tumour cells has been used as a predictive biomarker for response to next-generation AR-targeting agents with patients demonstrating a positive correlation between AR-V expression and compromised drug efficacy [8].

The mechanisms that regulate AR-V generation in cells are well characterised with splicing aberrations and genomic deletion of exons 4–8, that encode the LBD, being largely responsible for the production of receptor variants in CRPC [27]. However, our understanding of regulatory processes that govern the functionality of AR-Vs in disease is limited, particularly with respect to transcriptional requirements of the variants for driving the androgenic signalling cascade in advanced PC. Using FL-AR as a paradigm of multimodal transcriptional control, in which pioneer factors regulate chromatin deposition and activity of the receptor [14, 15], we focussed our study on the requirement of FOXA1 for AR-V activity in models of CRPC. Given that all reported AR-Vs contain the DNA-binding domain (DBD), that has been shown to be the site of FOXA1 interaction [37], combined with our initial findings that AR-Vs were constitutively chromatin-bound irrespective of hormonal/anti-androgen status (Figure 1), suggested that FOXA1 may co-operate with receptor variants to control their transcriptional output. Using FOXA1 siRNAs, we found that depletion of the pioneer factor elevated expression of *PSA* and *KLK2* in CWR22Rv1 cells in both the presence and absence of DHT and Enzalutamide, and this was driven by up-regulated AR-V expression, specifically AR-V7 and AR-1/2/3/2b. Using a combination of ChIP and quantitative mRNA analyses, we identified the existence of a negative feedback loop in CWR22Rv1 cells that is controlled by FOXA1 and acts to limit expression of AR-Vs. Depletion of FOXA1 attenuates binding of receptor variants to a downstream repressor element in intron 2 of the *AR* gene and hence selectively up-regulates expression of AR-Vs and, as a consequence, *PSA* and *KLK2*. Up-regulation of these genes has been recently reported in FOXA1-depleted LNCaP cells grown in the absence of androgens that occurs, in part, as a result of up-regulated FL-AR expression and chromatin association at these genomic loci [19]. Moreover, global AR chromatin association was found to be reconfigured in the absence of FOXA1 to new AR binding sites (ARBSs), suggesting the pioneer factor may be repressive to FL-AR binding at discriminate *cis*-regulatory elements [19]. Our analyses of FOXA1 knockdown in LNCaP cells has confirmed some of these findings, but intriguingly, we have demonstrated that up-regulated *PSA* and *KLK2* expression is insensitive to Enzalutamide and could be a result of elevated AR-V7, as well as FL-AR, expression (Supplementary Figure S15). It will be interesting to extrapolate these initial findings to include ChIP-Seq studies to interrogate the contribution, if any, of AR-Vs to the new FOXA1-independent ARBSs in LNCaP cells identified in Jin *et al*., (2014) [19].

A key observation from our CWR22Rv1 experiments was of selective up-regulation of AR-V7 and AR-1/2/3/2b, but not FL-AR, in response to FOXA1 knockdown (Figure 3), suggesting potential interplay between the splicing machinery and pioneer factor activity. A recent report has demonstrated that the binding of ASF2 and U2AF65 to splicing enhancer elements adjacent to exon 3b of the *AR* gene regulates the generation of AR-V7 in both VCaP cells, and the androgen-independent LNCaP cell derivative LN95, in ADT conditions [33]. Importantly, depletion of these splicing factors down-regulated AR-V7 levels without impacting on FL-AR mRNA indicating that discriminate spliceosomal function can be attributed to the processing of AR-Vs in models of CRPC. We speculate that flux to splicing factor expression and/or activity in response to FOXA1 knockdown could account for elevation of AR-Vs in CWR22Rv1 cells. Intriguingly ASF2 and U2AF65 mRNA was reduced upon pioneer FOXA1 depletion in our experiments (data not shown) suggesting that other splicing factors may contribute to AR-V production in these cells and this is being interrogated in our on-going studies.

Expression analysis demonstrated that 41% of AR-V target genes were also co-regulated by FOXA1 indicating a considerable overlap and potential dependency of the receptor variants on pioneer factor activity for both AR-activated and—repressed transcriptomes. This was confirmed at several target genes, including *UBE2C*, *NKX3.1, ATAD2* and *CCNA2*, in which depletion of either AR, AR-Vs or FOXA1 in Enzalutamide-treated CWR22Rv1 cells down-regulated their expression. Critically, knockdown of FOXA1 attenuated enrichment of AR-Vs to *cis*-regulatory elements of *UBE2C* and *CCNA2*, indicating that the pioneer factor is required to facilitate constitutive receptor variant deposition at distinct genomic loci akin to the control of DHT-stimulated FL-AR recruitment to target genes in PC cells [16, 19]. We identified an 80% overlap between our AR-V transcriptome and a 25 gene signature reported by Hu *et al*., (2012) [34] to be regulated specifically by ectopically-expressed AR-V7 in LNCaP cells, including *UBE2C* that is an established AR-V target gene [34]. Moreover, approximately 52% of these overlapping genes were also regulated by FOXA1 in our data-set, suggesting considerable interplay between the receptor variants and FOXA1 for transcriptional output. To further assess FOXA1 function in CRPC, we compared our AR-V-target gene signature with that from a recent study in which an AR-V transcriptome was identified by subtractive analysis of total AR- and FL-AR-dependent gene-sets in CWR22Rv1 cells [35]. Consistent with the role of FOXA1 as a regulator of AR-Vs, we found that almost 50% of the overlapping AR variant-target genes from our study and the one performed by Lu *et al*., (2015) [35] were also controlled by FOXA1. Importantly, however, we found only 20% concordance between the two independent AR-V target gene signatures that is likely to be a reflection of distinct, but valid experimental procedures that have been applied to delineate a specific AR-V transcriptional programme in the complex background of FL-AR expression. In contrast to selective FL-AR knockdown [35], we used steroid-depleted media supplemented with Enzalutamide to down-regulate FL-AR signalling and applied total AR knockdown to identify AR-V-driven genes. Differences in siRNA oligonucleotides as well as duration and efficacy of knockdown (48 hours in this study versus 72 hours in [35]) is an important distinction that could promote significant variation in the two experimental outputs.

There remains a paucity of agents/drugs available to attenuate receptor variant activity in the clinical setting hence components of the AR-V signalling network could represent novel therapeutic targets. The recent identification of the anti-helminthic drug Niclosamide as a selective destabiliser of AR-Vs in CWR22Rv1 cells and an AR-V7 expressing C4–2B cell line derivative suggests that selective targeting of these aberrantly functioning receptors is achievable at least in pre-clinical studies [38]. Consistent with this notion of blocking AR-V function, EPI-001 covalently binds to the AR N-terminus and has been shown to down-regulate both FL-AR- and AR-V-mediated signalling *in vitro* and *in vivo* [39]. Although a recent report has defined off-target effects of EPI-001 outside of the AR signalling cascade in cell line models of disease [40], there remains major scope to utilise more refined agents targeting the unstructured transactivation domain of the receptor as a strategy for abrogating global AR function in advanced disease. We have shown that FOXA1 is a key regulator of CWR22Rv1 proliferation, in part, by controlling AR-V-driven expression of cell cycle regulated genes, including *CCNA2*, *CCNB1*, *CCNE2* and *CDK1* suggesting that the pioneer factor represents an additional tractable therapeutic target in AR-V-expressing CRPC. In support of this, several studies have demonstrated up-regulated FOXA1 protein in advanced disease and knockdown attenuates growth of additional PC cell lines [16, 41]. Outside of PC, FOXA1 has been demonstrated to be a vital regulator of ER signalling in breast cancer and therefore is a *bona fide* therapeutic target in this disease setting [15, 42]. Targeting FOXA1 will be challenging, but inhibitors of an additional AR pioneer factor GATA2 are available and have been successfully applied to several cell-line-based disease models, including PC [43]. In all, our data supports the concept that AR-V function is controlled by FOXA1 and provides additional justification for targeting pioneer factor activity in advanced CRPC.

## MATERIALS AND METHODS

### Transient siRNA transfection and cell treatments

CWR22Rv1, LNCaP and VCaP cells were maintained in RPMI1640 media (Sigma) supplemented with 10% foetal calf serum (FCS) and 5% L-glutamine at 37°C. Transient transfection of FOXA1 and AR siRNAs (listed in Supplementary Table S5) was performed using Lipofectamine RNAiMax (Life Sciences). For 10 nM DHT, 1 μM Bicalutamide and 1 μM Enzalutamide treatments, cells were grown in phenol red-free RPMI1640 (Life Sciences) containing 10% steroid-depleted FCS (Hyclone) for at least 24 hours prior to addition of compounds.

### Quantitative PCR, western analyses, immunoprecipitation and chromatin extraction

Quantitative PCR was used to assess expression of AR-target genes (see Supplementary Table S3 for primer sequences) using cDNA generated from Trizol-mediated RNA extractions as described in [12]. Data represents the average of three independent experiments performed in triplicate. Western blotting was performed as described in [44] using the following antibodies; AR (N-20), AR (C-19), FOXA1, PARP-1 (Santa Cruz Biotechnology), AR-V7 (Precision Antibodies) and α-Tubulin (Sigma). Immunoprecipitation was conducted as described in [12] using the AR-V7 antibody. For chromatin preparation, CWR22Rv1 cells grown in the presence and absence of 10 nM DHT, 1 μM Bicalutamide/1 μM Enzalutamide or transiently transfected with FOXA1 siRNAs, were subject to the extraction protocol described in [44].

### Chromatin immunoprecipitation (ChIP) and FAIRE analyses

ChIP assays were performed as described in [44] utilising AR (N-20), AR (C-19) and FOXA1 antibodies (Santa Cruz Biotechnology). Quantitative PCR of resultant ChIP'd DNA was performed using primers to *AR* intron 2 and AR-target genes (see Supplementary Table S3 for sequences). For ChIP experiments investigating recruitment of AR and FOXA1 to target genes in response to FOXA1 depletion, CWR22Rv1 cells grown in steroid-depleted media were transfected with either siFOXA1 or siScr oligonucleotides for 48 hours prior to 24 hour stimulation with 1 μM Enzalutamide or vehicle control prior to chromatin preparation. Data is presented as the mean of at least 2 independent experiments. FAIRE was performed in both LNCaP and CWR22Rv1 cells grown in steroid-depleted media for 48 hours prior to 10 nM DHT treatment for 4 hours and formaldehyde cross-linking for 7 minutes. Cells were lysed and chromatin sonicated using the same ChIP protocol as above. Post-sonication, 2 × phenol/chloroform extractions were performed before a final chloroform-isoamyl alcohol (24:1) vortex and spin at 12,000 g for 5 minutes. 1/10th volume of a 3 M sodium acetate (pH 5.2)/20 mg/ml glycogen mixture was added to supernatants together with 2 × volume of 100% ethanol to precipitate DNA at −20°C overnight. DNA was pelleted by centrifugation at 12,000 g for 30 minutes at 4°C, washed in 70% ethanol and air dried prior to resuspension in ChIP elution buffer (see [12]) and DNA clean-up using GeneElute mammalian genomic DNA miniprep kits (Sigma). Resultant DNA was analysed by quantitative PCR using primers listed in Supplementary Table S3. All statistical testing was performed using a student *T*-test in Microsoft Excel with * denoting *p* < 0.05.

### Cell proliferation and flow cytometry

CWR22Rv1 cells grown in steroid-depleted conditions +/− 10 nM DHT and 1 μM Enzalutamide post siFOXA1 and siAR transfection were trypsinised and counted individually using a haemocytometer. Data represents three independent experiments performed in quadruplicate. Cell cycle analysis using propidium iodide flow cytometry was performed as described in [45]. All statistical testing was performed using a student *T*-test in Microsoft Excel with * denoting *p* < 0.05.

### Micro-array analysis

RNA was extracted from CWR22Rv1 cells grown in steroid-depleted media subjected to control, AR or FOXA1 knockdown for 24 hours prior to vehicle or 1 μM Enzalutamide treatment for 24 hours. Samples were hybridised onto an Illumina HT12 v4 BeadChip Array (performed by The Wellcome Trust Centre for Human Genetics, Oxford University. Array processing, normalisation and quality control checks were performed using the R package ‘Lumi’. Probes intensity values were converted to VSD (variance stabilised data) using variance stabilizing transformation. The robust spline normalisation (RSN) was used as the array normalisation method. Outlier samples, poor quality probes (detection threshold < 0.01), and probes that are not detected at all in the remaining arrays were removed prior downstream analysis. The remaining probe (22,551) normalised intensity, VSD was used in the differential expression analysis. Differential expression analysis was performed using the R package ‘Limma’, and *P* values were adjusted to control the false discovery rate (FDR) using the Benjamini–Hochberg method. Gene ontology analysis was performed as described in [45].

## SUPPLEMENTARY DATA FIGURES AND TABLES






