# Supplementary material for: FOXA1 regulates androgen receptor variant activity in models of castrate-resistant prostate cancer
Source: Oncotarget. 2015 Aug 13;6(30):29782–94. doi: 10.18632/oncotarget.4927 (PMC4745762; doi:10.18632/oncotarget.4927)
Supplement: Supplementary file 1 [file oncotarget-06-29782-s001.pdf]

SUPPLEMENTARY DATA

REFERENCES

1. Hu R, Lu C, Mostaghel EA, Yegnasubramanian S, Gurel M, Tannahill C, Edwards J, Isaacs WB, Nelson PS, Bluemn E, Plymate SR, Luo J. Distinct transcriptional programs mediated by the ligand-dependent full-length androgen receptor and its splice variants in castration-resistant prostate cancer. Cancer Res. 2012; 72:3457–3462.
2. Lu J, Lonergan PE, Nacusi LP, Wang L, Schmidt LJ, Sun Z, Van der Steen T, Boorjian SA, Kosari F, Vasmatzis G, Klee GG, Balk SP, Huang H, Wang C, Tindall DJ. The cistrome and gene signature of androgen receptor splice variants in castration resistant prostate cancer cells. J Urol. 2015; 193:690–698.

SUPPLEMENTARY FIGURES AND TABLES

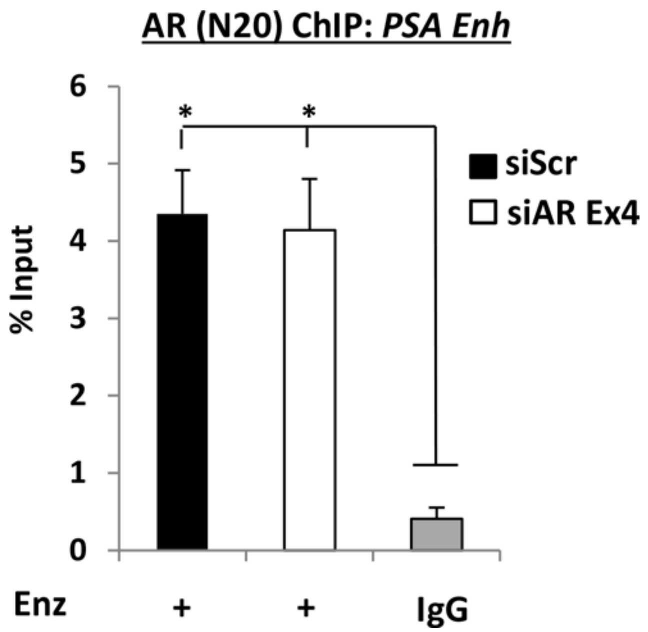

Supplementary Figure S1: CWR22Rv1 cells were transiently transfected with either scrambled (siScr) or FL-AR-targeting Exon 4 siRNAs for 48 hours prior to 24 hour Enzalutamide treatment and ChIP analysis using AR N20 and control antibodies. Enrichment of AR-Vs was assessed at the enhancer element of the *PSA* gene. Data represents the mean of three independent experiments +/− SE (* denotes p-value < 0.05).

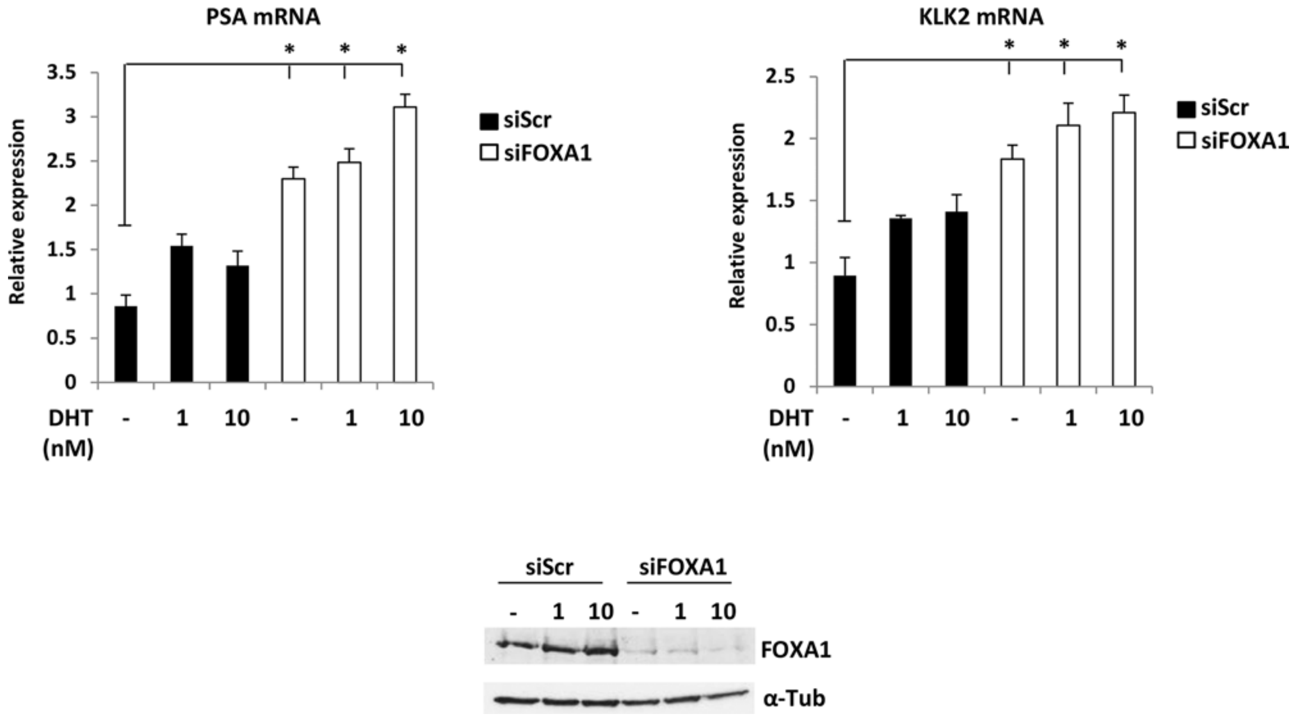

Supplementary Figure S2: CWR22Rv1 cells depleted of FOXA1 by siRNA for 24 hours were treated with either 1 or 10 nM dihydrotestosterone (DHT) for an additional 24 hours prior to RNA extraction and quantitative analysis of *PSA* and *KLK2* expression. Immunoblotting confirmed depletion of FOXA1 by siRNA. Data represents the mean of three independent experiments +/− SE (* denotes p-value < 0.05).

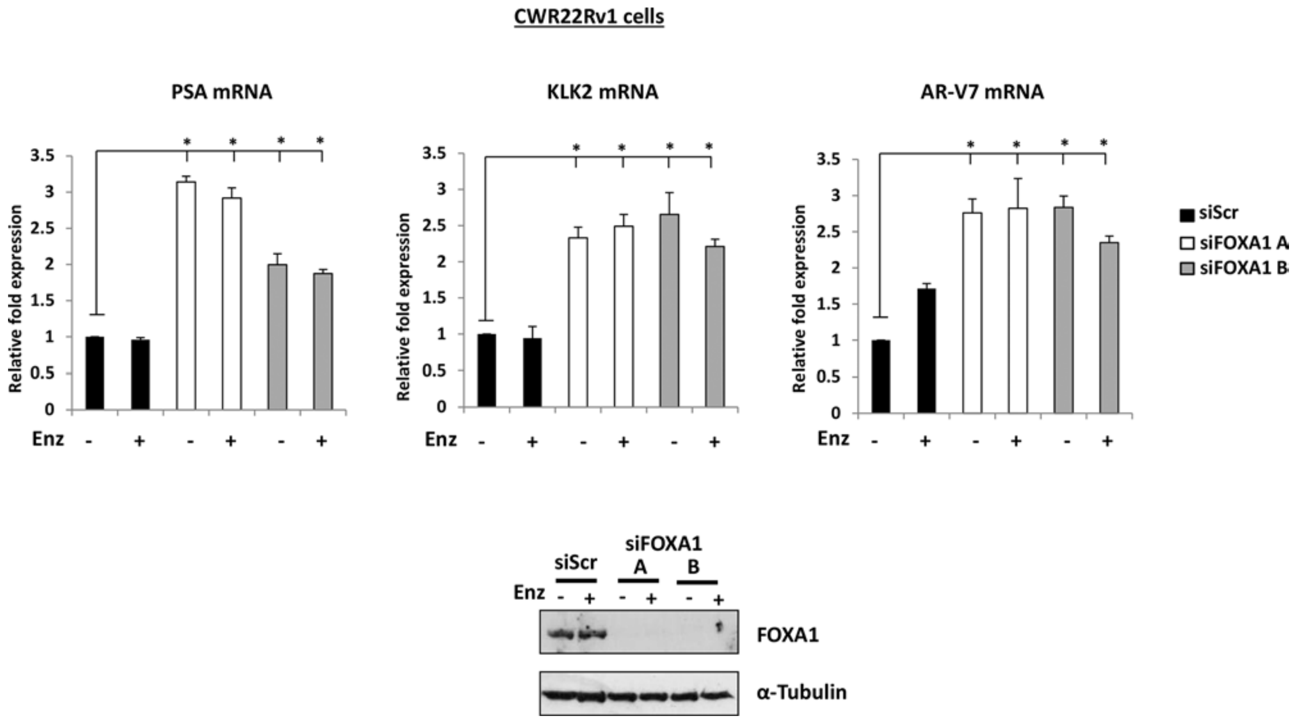

Supplementary Figure S3: FOXA1 was depleted by two individual siRNAs (siFOXA1 A. and B.) for 48 hours in CWR22Rv1 cells and levels of PSA, KLK2 and AR-V7 mRNA were measured after 24 hours 1 μM Enzalutamide treatment by quantitative PCR. Representative westerns of FOXA1 are shown in the lower panel. Data represents the mean of three independent experiments +/− SE (* denotes *p*-value < 0.05).

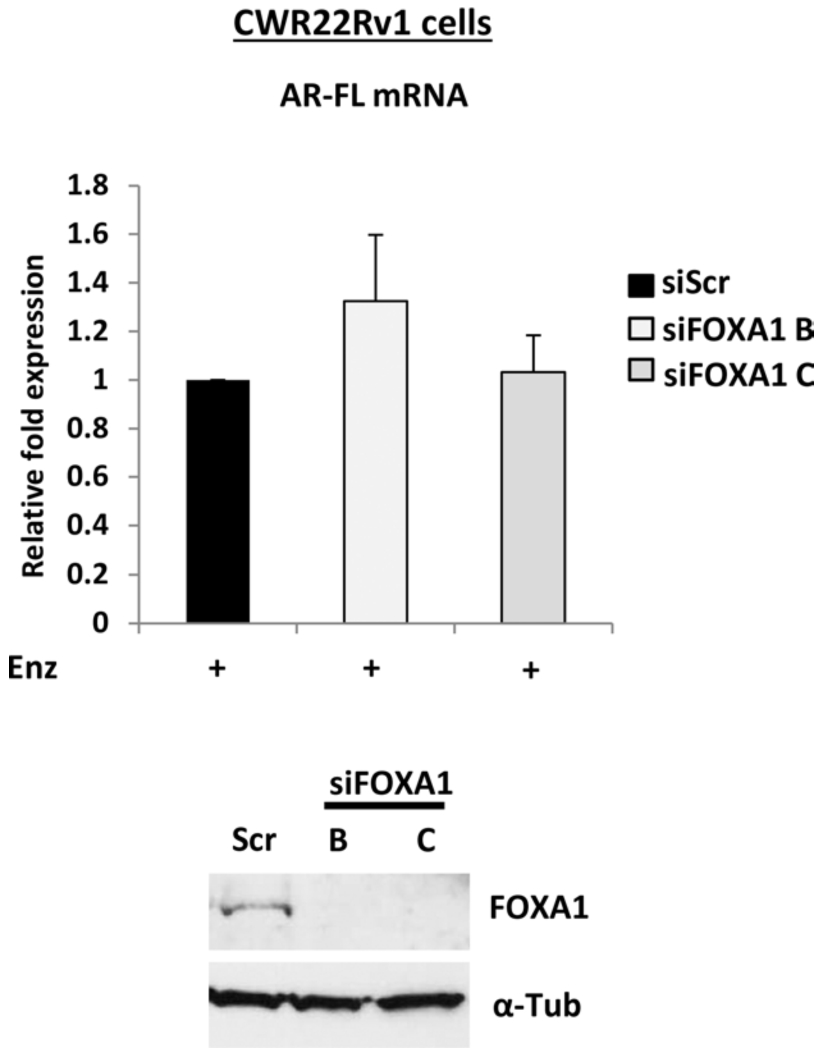

Supplementary Figure S4: FOXA1 knockdown in CWR22Rv1 cells was performed using two additional oligonucleotides (B and C) for 48 hours prior to 24 hour Enzalutamide treatment and RNA extraction. Full-length AR (AR-FL) expression was analysed by quantitative PCR. Data represents the mean of three independent experiments +/− SE (* denotes *p*-value < 0.05). Representative western blots to confirm FOXA1 depletion is shown.

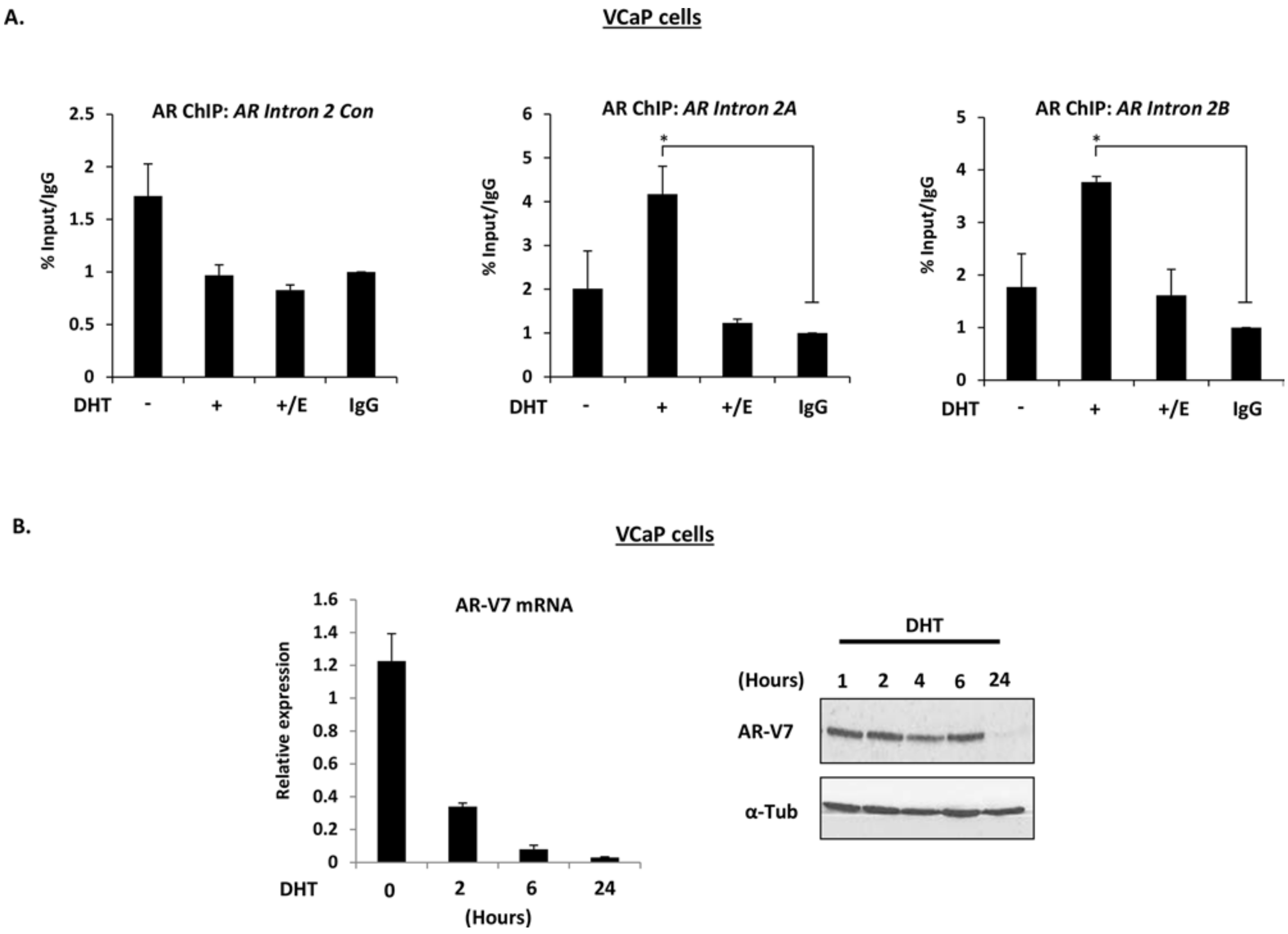

Supplementary Figure S5: A. Chromatin immunoprecipitation (ChIP) was performed using an AR (N20) antibody in VCaP cells grown in steroid-depleted media and treated with 10 nM DHT and/or 1 µM Enzalutamide for 4 hours to assess AR recruitment to the *AR* gene DRE. **B.** VCaP cells were grown in steroid-depleted media for 48 hours and then treated with 10 nM DHT for 0–24 hours prior to western analysis using anti-AR and -α-Tubulin antibodies. Data represents the mean of three independent experiments +/− SE (* denotes p-value < 0.05).

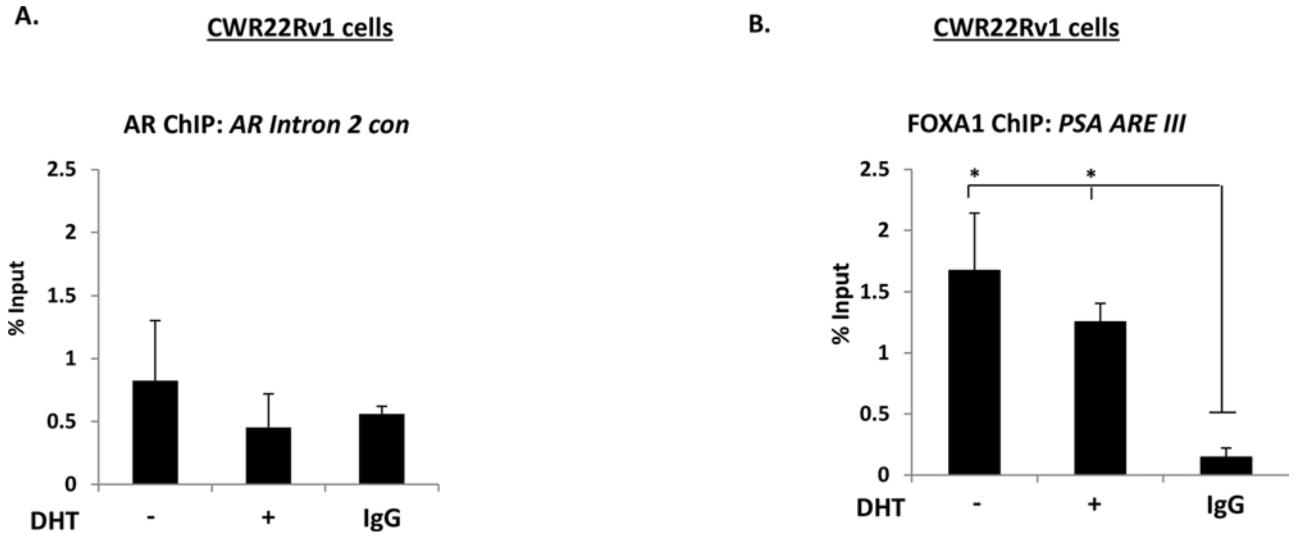

Supplementary Figure S6: A. Chromatin immunoprecipitation (ChIP) experiments in CWR22Rv1 cells using an AR (N20) antibody to assess recruitment of AR-Vs to a control region adjacent to intron 2 of the *AR* gene in response to 4 hour 10 nM DHT treatment. **B.** As in **(**A), but ChIP was conducted using an anti-FOXA1 antibody to assess recruitment of FOXA1 to the enhancer element of the *PSA* gene. Data represents the mean of three independent experiments +/− SE (* denotes p-value < 0.05).

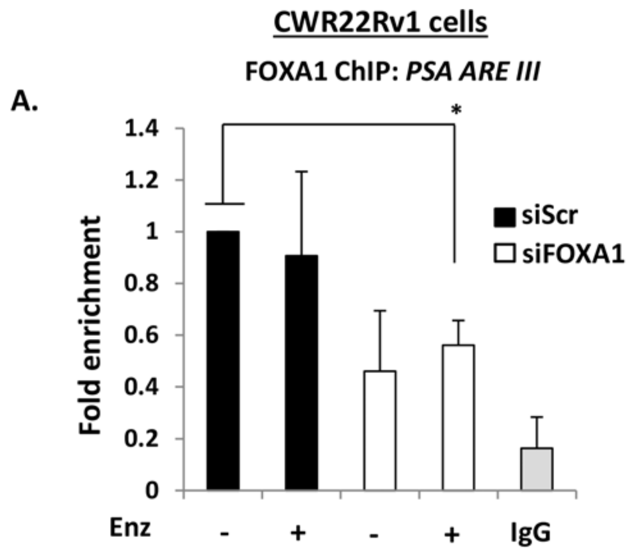

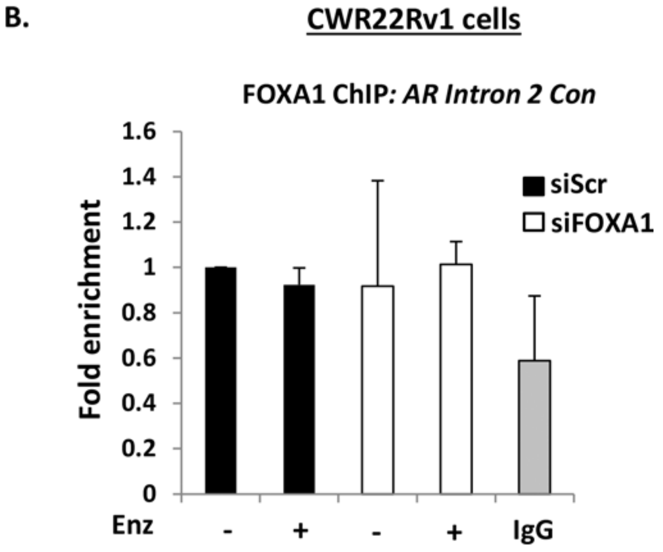

Supplementary Figure S7: CWR22Rv1 cells depleted of FOXA1 were treated with Enzalutamide for 4 hours and subject to ChIP using an anti-FOXA1 antibody to assess recruitment to either **A.** the *PSA* gene enhancer or **B.** a control region adjacent to intron 2 of the *AR* gene. Data represents the mean of three independent experiments +/− SE (* denotes *p*-value < 0.05).

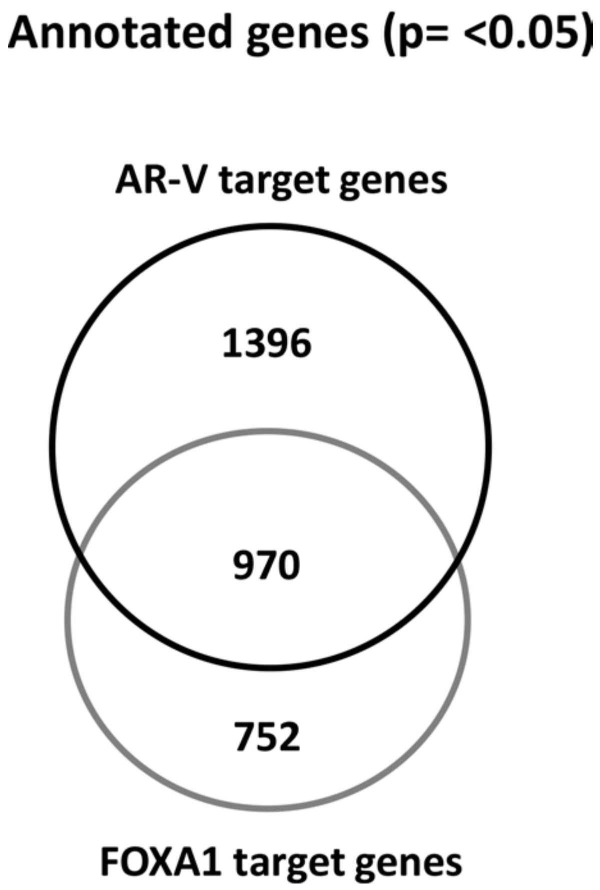

Supplementary Figure S8: Venn diagram of significantly de-regulated genes in response to AR and FOXA1 depletion in CWR22Rv1 cells grown in steroid-depleted conditions + 1 μM Enzalutamide.

A.

B.

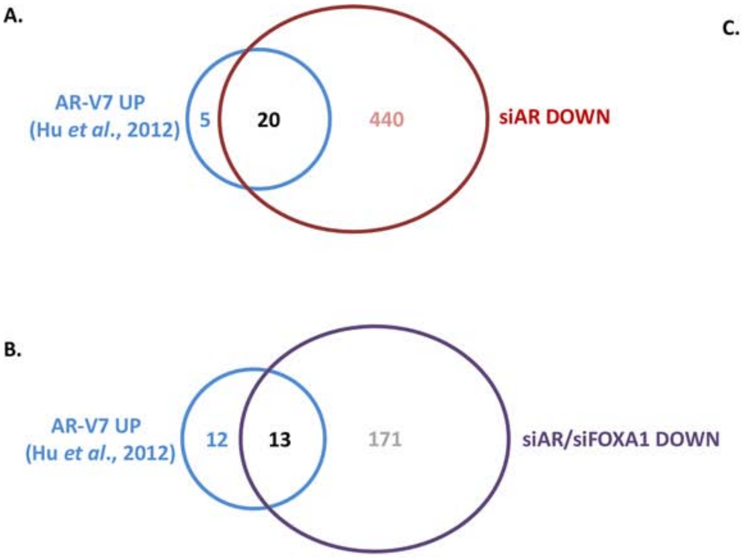

C.

Overlapping AR-V7 UP genes

Gene: siAR DOWN	Gene: siAR/siFOXA1 DOWN
BIRC5	*BIRC5*
BUB1	*BUB1*
KPNA2	*KPNA2*
CENPE	*CENPE*
ESPL1	*ESPL1*
NEK2	*NEK2*
CIT	*CIT*
CCNA2	*CCNA2*
CDC25C	*CDC25C*
KIF15	*KIF15*
UBE2C	*UBE2C*
NUSAP1	*NUSAP1*
BIRC5	*BIRC5*
CDCA5	
KNTC1	
BUB1B	
CDC25B	
MAD2L2	
KIF22	
MAD2L1	

Supplementary Figure S9: Venn diagrams showing overlap between the AR-V7 UP signature (Hu et al.,2012 (1)) and either our AR-V target gene-set **A.** or joint AR-V/FOXA1-regulated gene-set **B. C.** Table of overlapping genes shown in (A) and (B).

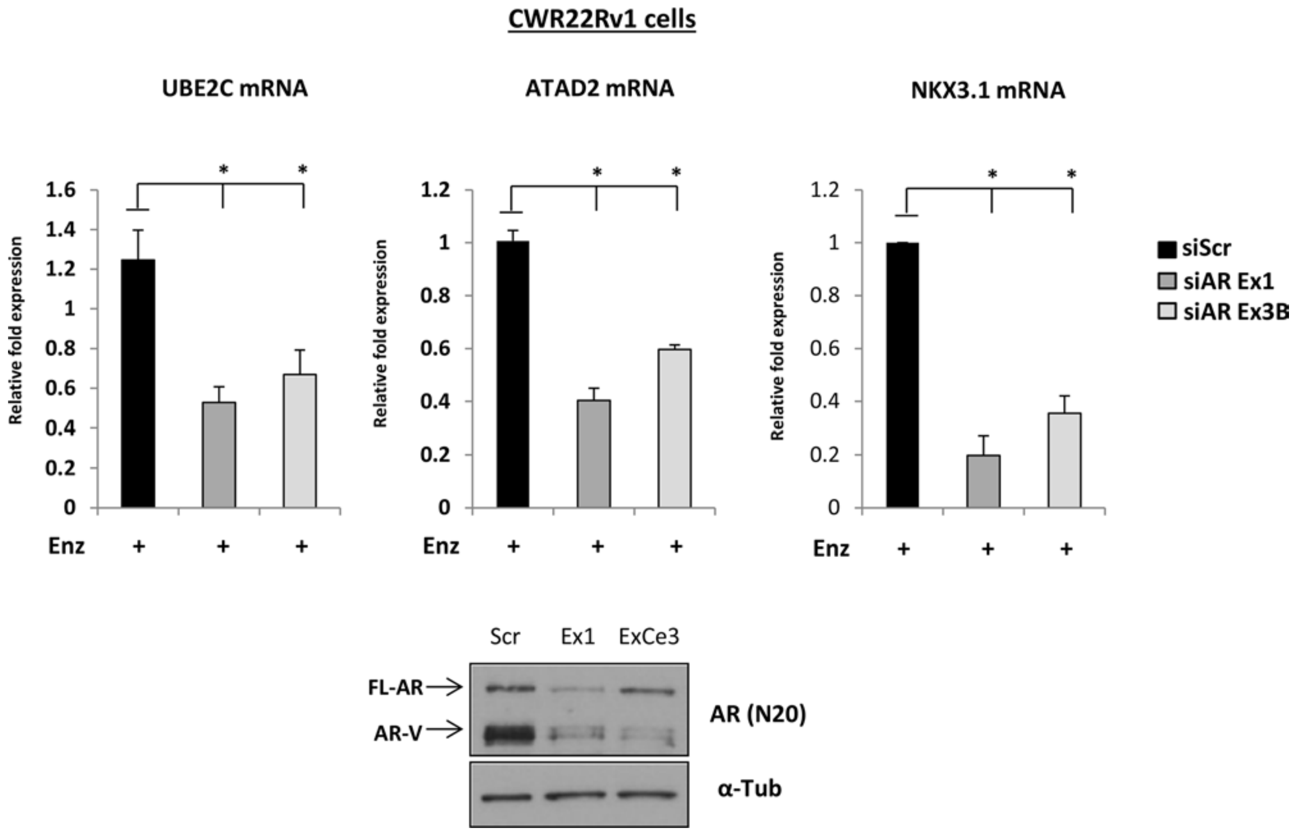

Supplementary Figure S10: CWR22RV1 cells were transiently transfected with either control (siScr), AR Exon 1-targeting or AR Exon 3B-targeting siRNAs to deplete all or AR-Vs, respectively, for 48 hours prior to 24 hour Enzalutamide treatment and RNA extraction. *UBE2C*, *ATAD2* and *NKX3.1* expression was analysed by quantitative PCR. Data represents the mean of three independent experiments +/− SE (* denotes p-value < 0.05). Representative western blots are shown to demonstrate selective depletion of AR species.

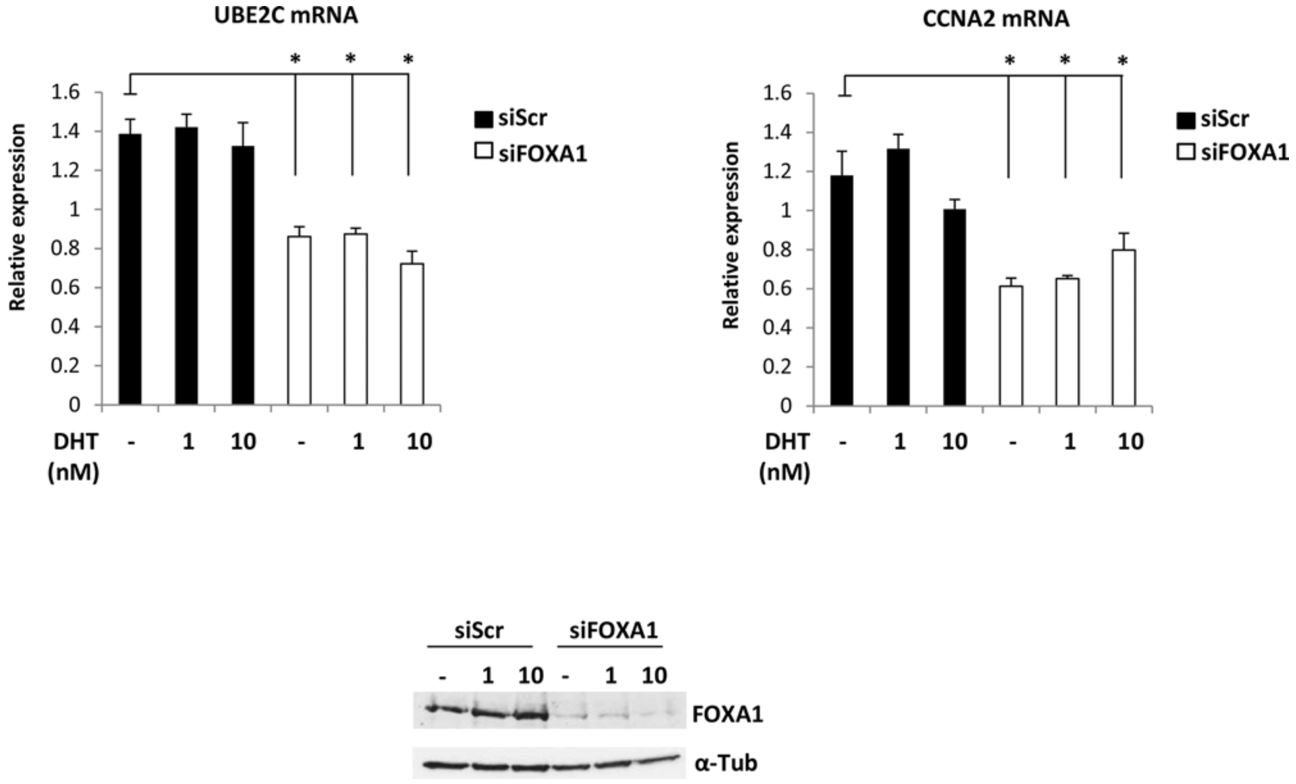

Supplementary Figure S11: CWR22Rv1 cells depleted of FOXA1 by siRNA for 24 hours were treated with either 1 or 10 nM dihydrotestosterone (DHT) for an additional 24 hours prior to RNA extraction and quantitative analysis of *UBE2C* and *CCNA2* expression. Immunoblotting confirmed depletion of FOXA1 by siRNA. Data represents the mean of three independent experiments +/− SE (* denotes p-value < 0.05).

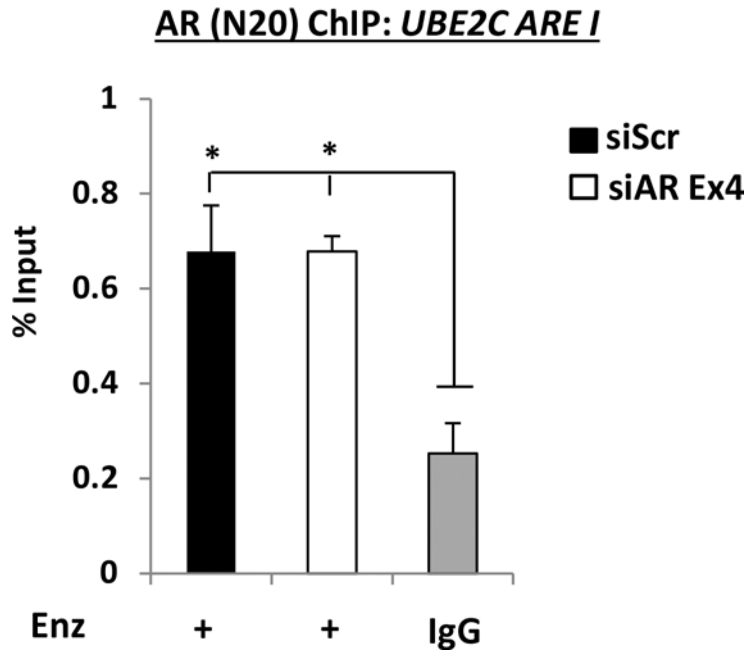

Supplementary Figure S12: CWR22RV1 cells depleted of AR-FL by AR Exon 4-targeting siRNAs for 48 hours prior to 24 hour Enzalutamide treatment were subject to chromatin immunoprecipitation using AR N20 and control antibodies. Enrichment of AR-Vs to the cis-regulatory element of *UBE2C* was assessed by quantitative PCR. Data represents the mean of three independent experiments +/− SE (* denotes p-value < 0.05).

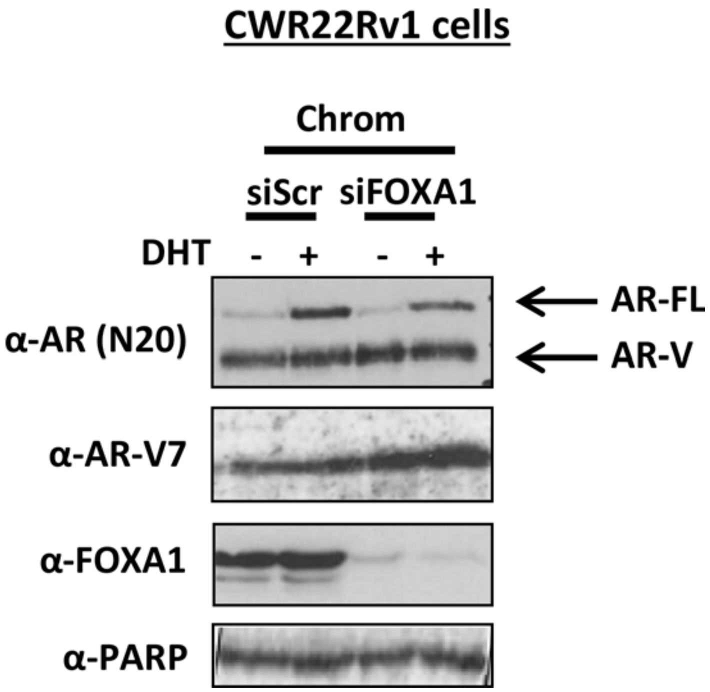

Supplementary Figure S13: CWR22Rv1 cells depleted of FOXA1 were treated with 10 nM DHT for 4 hours prior to chromatin fractionation and western blotting using anti-AR, -AR-V7, -FOXA1 and -PARP1 antibodies.

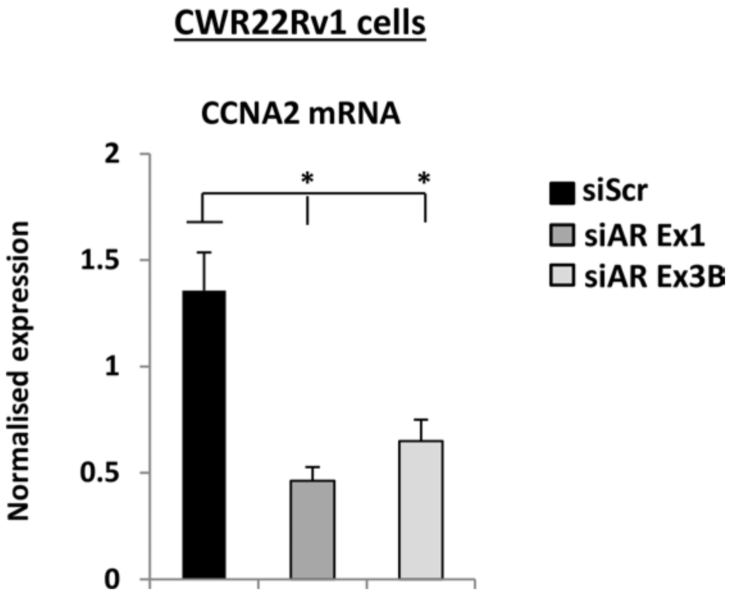

Supplementary Figure S14: CWR22RV1 cells were transiently transfected with either control (siScr), AR Exon 1-targeting or AR Exon 3B-targeting siRNAs to deplete all or AR-Vs, respectively, for 48 hours prior to 24 hour Enzalutamide treatment and RNA extraction. CCNA2 expression was analysed by quantitative PCR. Data represents the mean of three independent experiments +/− SE (* denotes p-value < 0.05).

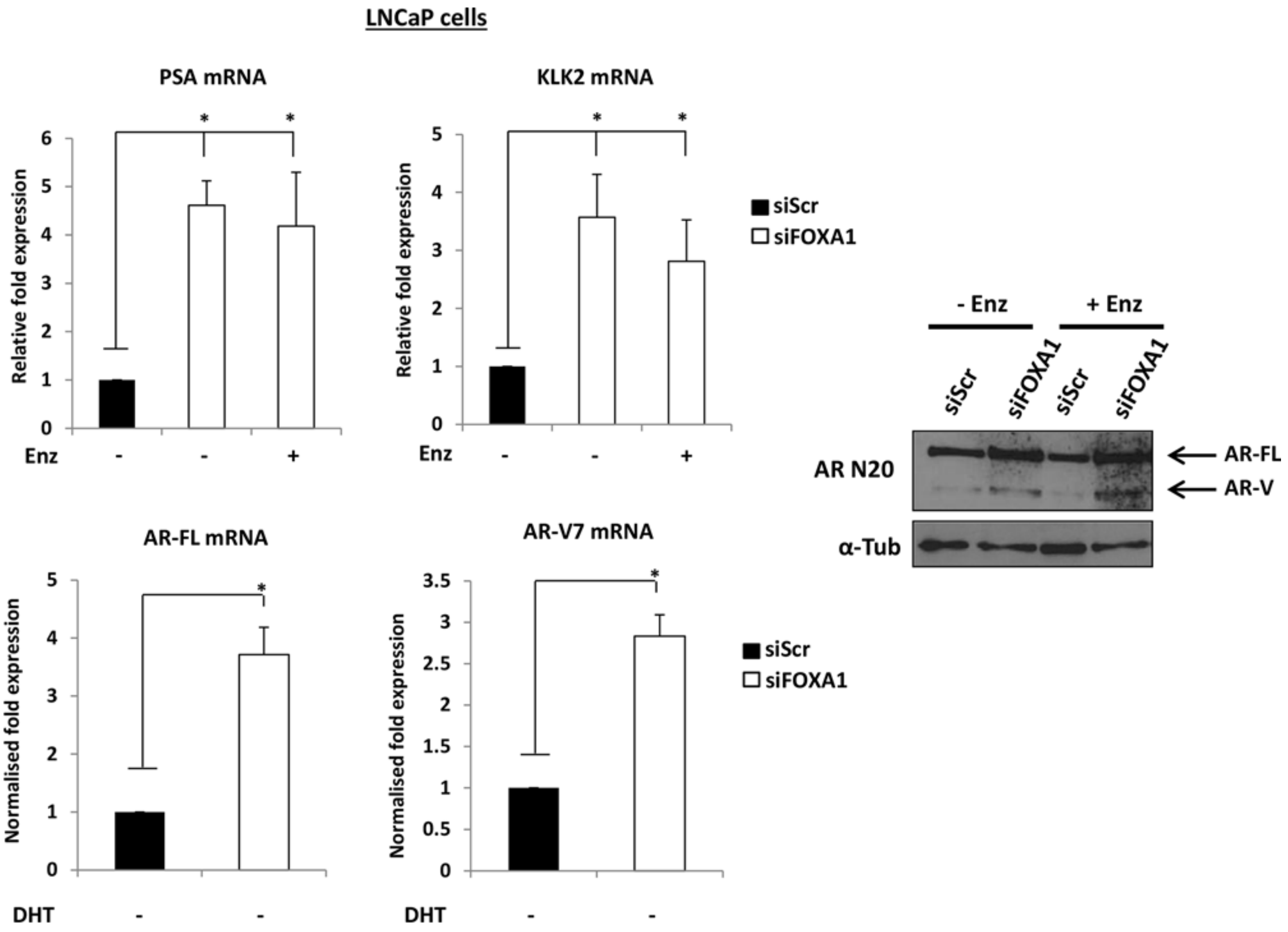

Supplementary Figure S15: FOXA1 levels were reduced by siRNA knockdown for 48 hours in LNCaP cells and mRNA levels of PSA, KLK2, FL-AR and AR-V7 was measured after 24 hours 10 nM DHT or 1 µM Enzalutamide stimulation by quantitative PCR. Representative western blots using anti-AR and –α-Tubulin antibodies is shown. Data represents the mean of three independent experiments +/− SE (* denotes *p*-value < 0.05).

Supplementary Table S1: List of AR-V target genes

Supplementary Table S2: List of FOXA1 target genes

Supplementary Table S3: Overlapping AR-V-target genes with Lu *et al*., (2015)(2)

Gene

ABTB1	DNAJC12	HSPB6	MT1G	SELENBP1
ACADVL	DYNLT3	ICAM3	MT2A	SFXN1
ACOT7	EFNB2	IGFBP3	NFKBIZ	SLC40A1
ACPP	EFNB3	IMMP2L	PLA2G3	SLCO2A1
ADD3	FHDC1	ING2	PMEPA1	SNORD68
APPL2	FSCN1	LAMB2	PPP2R2A	TAX1BP1
ARMCX3	G3BP2	LEF1	PRELID1	TSPAN3
ATP5I	GADD45A	LXN	RAB11A	ZNF789
BAMBI	GALNT4	MICAL1	RAB9A	ZSWIM4
BOLA3	GLDN	MKNK2	RNY4	
CCNDBP1	GNG4	MT1A	RPL34	
CCNG2	GPC4	MT1E	SDC4	

Supplementary Table S4: Overlapping AR-V/FOXA1-target genes with Lu *et al*., (2015)(2)

Gene

ABTB1	GPC4	MT1G
ACADVL	IGFBP1	NFKBIZ
ATP5I	IGFBP3	PLA2G3
BOLA3	ING2	RNY4
CCNDBP1	LAMB2	RPL34
EFNB2	LEF1	SDC4
FSCN1	LXN	SELENBP1
G3BP2	MKNK2	SFXN1
GNG4	MT1A	SLCO2A1
		TSPAN3

Supplementary Table S5: Primer and siRNA sequences

Oligo Name	Sequence (5′-3′)
siFOXA1 A	GAGAGAAAAAAUCAACAGC
siFOXA1 B	GCACUGCAAUACUCGCCUU
siAR	CCAUCUUUCUGAAUGUCCU
FOXA1 mRNA F	GAAGATGGAAGGGCATGAAACCA
FOXA1 mRNA R	TGGCATAGGACATGTTGAAGGACG
FL-AR mRNA F	CATGTGGAAGCTGCAAGGTCT
FL-AR mRNA R	TCTGTTTCCCTTCAGCGGC
AR exon 3 F mRNA	AACAGAAGTACCTGTGCGCC
AR-V7 R mRNA	TCAGGGTCTGGTCATTTTGA
AR-1/2/3/2b R mRNA	GTTCATTCTGAAAAATCCTTCAGC
PSA F mRNA	GGTGCATTACCGGAAGTGGAT
PSA R mRNA	TGGTCATTTCCAAGGTTCCAA
KLK2 F mRNA	AGCATCGAACCAGAGGAGTTCT
KLK2 R mRNA	TGGAGGCTCACACACCTGAAGA

Oligo Name	Sequence (5′-3′)
UBE2C mRNA F	TGCCCTGTATGATGTCAGGA
UBE2C mRNA R	GGGACTATCAATGTTGGGTTCT
NKX3.1 mRNA F	AGCCAGAAAGGCACTTGGG
NKX3.1 mRNA R	GGCGCCTGAAGTGTTTTCA
CCNA2 mRNA F	GAAGACGAGACGGGTTGCA
CCNA2 mRNA R	AGGAGGAACGGTGACATGCT
PSA Enh ChIP F	TGGGACAACTTGCAAACCTG
PSA Enh ChIP R	CCAGAGTAGGTCTGTTTTCAATCCA
TMPRSS2 FAIRE F	TGGTCCTGGATGATAAAAAAAGTTT
TMPRSS2 FAIRE R	GACATACGCCCCACAACAGA
UBE2C ARE I ChIP F	TGCCTCTGAGTAGGAACAGGTAAGT
UBE2C ARE I ChIP R	TGCTTTTTCCATCATGGCAG
UBE2C ARE 2 ChIP F	CCACAAACTCTTCTCAGCTGGG
UBE2C ARE 2 ChIP R	TTCTTTCCTTCCCTGTTACCCC

Oligo Name	Sequence (5′-3′)
CCNA2 ChIP F	TTAGTGAGCTGTCCAGTGACTCAAT
CCNA2 ChIP R	CCCATGTATTAAAGTAGCTTCTGTAAACA
AR Exon 2 Con ChIP F	GTGCTGTACAGGAGCCGAAG
AR Exon 2 Con ChIP R	AACTTCACCGAAGAGGAAAGG
AR Exon 2 2A ChIP F	CCATCATGTGCATTATGTGC
AR Exon 2 2A ChIP R	CCAAACAGCACTCCATGTGT
AR Exon 2 2B ChIP F	CACATGGAGTGCTGTTTGGT
AR Exon 2 2B ChIP R	GTAAACATCAGTGAGGATGGTG
